# Ancestral range reconstruction of remote oceanic island species of *Plantago* (Plantaginaceae) reveals differing scales and modes of dispersal

**DOI:** 10.1111/jbi.13525

**Published:** 2019-03-15

**Authors:** N. Iwanycki Ahlstrand, B. Verstraete, G. Hassemer, S. Dunbar‐Co, R. Hoggard, H. M. Meudt, N. Rønsted

**Affiliations:** ^1^ Natural History Museum of Denmark University of Copenhagen Copenhagen Denmark; ^2^ Natural History Museum University of Oslo Oslo Norway; ^3^ The Nature Conservancy Kaunakakai Hawaii USA; ^4^ Department of Microbiology and Plant Biology University of Oklahoma Tulsa Oklahoma USA; ^5^ Museum of New Zealand Te Papa Tongarewa Wellington New Zealand

**Keywords:** biogeographical range reconstruction, bird dispersal, island taxa, long‐distance dispersal, oceanic islands, *Plantago*

## Abstract

**Aim:**

The aim of this study was to resolve the phylogenetic placement of island taxa, reconstruct ancestral origins and resolve competing hypotheses of dispersal patterns and biogeographical histories for oceanic island endemic taxa within subgenus *Plantago* (Plantaginaceae).

**Location:**

Juan Fernández Islands, the Auckland Islands, Lord Howe Island, New Amsterdam Island, New Zealand, Tasmania, Falkland Islands, Rapa Iti and the Hawaiian Islands.

**Taxon:**

Island endemics within *Plantago* (Plantaginaceae), a globally distributed taxonomic group comprising approximately 250 species.

**Methods:**

We use Bayesian phylogenetic and divergence time analyses and historical biogeographical analysis of molecular sequence data to infer the ancestral origins of the oceanic island species in *Plantago*.

**Results:**

Taxa within subgenus *Plantago* form clades based on geographic proximities and challenge previous phylogenetic relationships and classification based on morphology. We infer that biogeographic histories of oceanic island taxa from multiple islands were shaped by dispersal at different scales and possibly by different types of birds. The highly remote Hawaiian Islands and Rapa Iti were colonized from North American taxa in a pattern corresponding to known migration routes of large marine birds, rather than from New Zealand as previously hypothesized. The island endemics of Juan Fernández, the Falkland Islands, Lord Howe, Auckland Islands and New Zealand are found to have sources in the nearest continental areas. The analyses confirm recent speciation within subgenus *Plantago* – which is particularly heightened in island lineages in Hawaii and Rapa Iti – but show slightly older divergence times than previous molecular dating studies.

**Main conclusions:**

Using molecular data to infer ancestral ranges for plants with uncertain taxonomic relationships can greatly improve our understanding of biogeographical histories and help elucidate origins, dispersal modes and routes in widespread lineages with complex distribution patterns such as *Plantago*. We improve understanding of important floristic exchange areas between continents and islands as a result of long‐distance dispersal. We infer that a combination of both stepping stone dispersal and extreme long‐distance dispersal can shape insular floras, and that multiple floristic areas can be the sources of closely related island taxa. However, despite the successful dispersal of *Plantago*, radiation in island archipelagos is generally limited suggesting specific traits may limit diversification.

## 1 INTRODUCTION

For over a century, evolutionary biologists have investigated island taxa regarding their ancestral origins and evolutionary histories. The isolation and often young age of oceanic islands are considered excellent systems in which to study dispersal and speciation (Emerson, 2002; Losos & Ricklefs, 2009). In recent decades, owing to the advancement of molecular phylogenetic and divergence analyses, evidence has been increasing that long‐distance dispersal (LDD) events by wind, oceanic drift and animal migrations play a larger role than originally thought in explaining widespread species distributions (de Queiroz, 2005; Gillespie et al., 2012; le Roux et al., 2014; Nathan, 2006; Nathan et al., 2008; Raxworthy, Forstner, & Nussbaum, 2002; Vences et al., 2003; Viana, Gangoso, Bouten, & Figuerola, 2016; Winkworth, Wagstaff, Glenny, & Lockhart, 2002). Long‐distance dispersal, rather than geological vicariance, regardless of the geological histories and ages of island systems, appears to be the principal driver of range evolution and subsequent speciation (Christenhusz & Chase, 2013; de Queiroz, 2005; Dupin et al., 2017; Gallaher, Callmander, Buerki, & Keeley, 2015; Givnish et al., 2004; Mitchell et al., 2016).

In the case of plants, the movement of propagules either internally or externally by birds is recognized as the most common type of LDD responsible for the distribution of disjunct plant lineages on remote oceanic islands, though dispersal by oceanic drift and weather events also play roles (Carlquist, 1966, 1967; Gillespie et al., 2012; Kistler et al., 2014; Nathan et al., 2008; Pole, 1994; Sanmartin & Ronquist, 2004). Birds are more often implicated as the main dispersal vectors for island plants based on an over‐representation of plant traits that favour bird dispersal in island taxa (Baldwin & Wagner, 2010; Carlquist, 1967; Gillespie et al., 2012). The nearest landmasses are often implicated as the primary sources for island taxa, yet, as a result of bird‐mediated LDD across extreme distances such as via the Pacific or Asia‐Australian bird flyways, thousands of kilometres can separate some of the most closely related taxa (Baldwin & Wagner, 2010; Gillespie et al., 2012). Thus, LDD events mediated by bird vectors can result in multiple theories of biogeographic origins for island plant lineages (Birch & Keeley, 2013; de Queiroz, 2005; Kainulainen, Razafimandimbison, Wikstrom, & Bremer, 2017; le Roux et al., 2014). Applying biogeographical models and using molecular phylogenetic relationships to reconstruct source areas for island taxa can help resolve complex biogeographical histories, identify important areas of floristic exchange and elucidate dispersal routes that shape island floras (Bacon, Simmons, Archer, Zhao, & Andriantiana, 2016; Bouckaert et al., 2014; Ho et al., 2015; Johnson, Clark, Wagner, & McDade, 2017; Matzke, 2014).

*Plantago* L., a genus of approximately 250 species, is a model group to study LDD processes and compare different hypotheses regarding dispersal modes and routes, due to its worldwide distribution, high dispersal capabilities and high number of single island endemic taxa (Dunbar‐Co, Wieczorek, & Morden, 2008; Hassemer, De Giovanni, & Trevisan, 2016; Rahn, 1996; Tay, Meudt, Garnock‐Jones, & Ritchie, 2010a). The genus is largely temperate in distribution, but occurs at high altitudes in tropical areas and on oceanic islands (van der Aart & Vulto, 1992). Previous phylogenetic and molecular divergence analyses have estimated that *Plantago s.l*. (including *Littorella* P.J.Bergius) diverged from its closest known relative, *Aragoa* Kunth, in the late Miocene to Pliocene, 7.1 million years ago (Ma) (Bello, Chase, Olmstead, Rønsted, & Albach, 2002; Rønsted, Chase, Albach, & Bello, 2002) or 2.8 Ma (Tay et al., 2010a). This finding of recent diversification of the genus rules out earlier hypotheses of vicariance in explaining the global distribution of the genus (i.e. as proposed by Rahn, 1996), and thus, long‐distance dispersal – presumably by birds – is the accepted scenario for *Plantago* (Dunbar‐Co et al., 2008; Rønsted et al., 2002; Tay et al., 2010a). Not only are the seeds of *Plantago* species known to be eaten by birds and other animals (Buse & Filser, 2014; Czarnecka & Kitowski, 2013; Panter & Dolman, 2012) but also *Plantago* species often grow alongside graminoids and other plants that are known to be typically eaten as fodder by birds prior to long migratory flights (Carlquist, 1966; Meudt, 2012; Rahn, 1996). Furthermore, dispersal in the genus is thought to be facilitated by the mucilaginous properties of wetted seeds, a specialized adaptation that assists with seed dispersal by increasing chance of seeds adhering to animals (Fischer et al., 2004; Rønsted et al., 2002; Tay et al., 2010a) or by attracting animals and facilitating dispersal via ingestion (Buse & Filser, 2014; Western, 2012). The mucilage in *P. lanceolata* L. seeds for example has been shown to be strongly adhesive to feathers and fur, especially when it has dried, which increases the potential of being transported externally over vast distances (Kreitschitz, Kovalev, & Gorb, 2016), while mucilage in *P. major* L. has been shown to attract invertebrates and facilitate endozoochory (Buse & Filser, 2014).

In addition to its adaptations to long‐distance dispersal, the majority of *Plantago* species have traits that likely assist in the successful colonization of new locations, including remote oceanic islands. For example, species are wind pollinated and many are self‐compatible – two important traits that are common in other island endemics (Carlquist, 1967; Rahn, 1996; Stuessy, Crawford, & Ruiz, 2018). Additionally, many *Plantago* island endemics grow on cliffs and other inaccessible habitats that may serve as refuges from natural threats, thereby facilitating successful establishment and survival in new areas (Dunbar‐Co, Sporck, & Sack, 2009; Stuessy et al., 2018).

The genus *Plantago* has been classified by Rahn (1996) into six subgenera, that is, *Plantago*,* Coronopus* (Lam. & DC.) Rahn, *Littorella* (P.J.Bergius) Rahn, *Bougueria* (Decne.) Rahn, *Psyllium* (Mill.) Harms & Reiche and *Albicans* Rahn; however, recent molecular phylogenetic studies have challenged classical taxonomic treatment. Subgenus *Albicans*, for example, has subsequently been included in subgenus *Psyllium* based on molecular phylogenetic studies (Rønsted et al., 2002), and subgenus *Littorella* was found to be sister to the remainder of *Plantago* and is most often considered a separate genus (Hassemer, Moroni, & O'Leary, 2018; Hoggard, Kores, Molvray, Hoggard, & Broughton, 2003; Kolář, 2014).

Subgenus *Plantago*, sensu Rahn (1996), is the largest of the subgenera with about 130 species currently recognized (Rahn, 1996; Rønsted et al., 2002). The subgenus is monophyletic (Hoggard et al., 2003; Rønsted et al., 2002; Tay et al., 2010a) and is distributed on all continents. This group is the focus of this work, as the subgenus includes the highest number of native *Plantago* species on oceanic islands, yet taxonomic, phylogenetic and biogeographic relationships between taxa in the subgenus remain some of the most poorly resolved, and morphological variation between many of the species is low (Meudt, 2012; Rahn, 1996; Rønsted et al., 2002). Previous molecular analyses have found Rahn's (1996) taxonomic sections within subgenus *Plantago* to be paraphyletic (Hoggard et al., 2003; Ishikawa, Yokoyama, Ikeda, Takabe, & Tsukaya, 2009; Tay et al., 2010a) though additional sampling is necessary to confirm this and improve our understanding of biogeographic histories.

Forty‐five taxa in subgenus *Plantago* have been described from 18 oceanic islands or island systems (Appendix I, Supporting Information). Several species are single island endemics to South Pacific Ocean islands or island archipelagos, Juan Fernández Islands off the coast of Chile, Galápagos Islands, Tonga Islands and Lord Howe Island and the Auckland Islands in Australasia. A single species is endemic to Madagascar, nine to mainland New Zealand, six to Tasmania, one species is native to both the Auckland Islands and Tasmania. Two species are endemic to St. Paul and New Amsterdam Islands, seven to New Guinea, two to Java, one species is recognized from Japan, and three species are recognized in Jeju. Three endemic species are currently recognized in the Hawaiian Islands, two species are endemic to Rapa Iti. In the South Atlantic Ocean, single endemic island species are known from Saint Helena, from Trindade Island and from the Falkland Islands.

The remoteness of the oceanic islands, the extreme distance between the nearest landmasses as well as uncertainty in the taxonomic relationships have resulted in numerous and often competing hypotheses as to the biogeographical histories of the island taxa in *Plantago* (Dunbar‐Co et al., 2008; Hoggard et al., 2003; Meudt, 2012; Rahn, 1996; Rønsted et al., 2002; Tay et al., 2010a, 2010b). Given adaptations of *Plantago* plants to LDD by birds, dispersal routes could be many, and relatedness between taxa may not reflect geographic proximity. For example, some of the island taxa (*P. rupicola*,* P. fernandezia*,* P. princeps s.l*. and *P. robusta*) exhibit typical island traits such as woody stems (Carlquist, 1970; Rahn, 1996; Stuessy et al., 2018), and based on such morphological similarities, *P. fernandezia* is thought to be more closely related to *P. princeps s.l*. in the Hawaiian Islands rather than to taxa in the Americas (Pilger, 1937; Rahn, 1996). However, the question remains whether plant propagules arrived in the Juan Fernández Islands from the western Pacific or from South America – the closest continental landmass where extant *Plantago* species are known (Rahn, 1996; Stuessy et al., 2018). Along similar lines, at least two competing hypotheses exist for the species endemic to the Hawaiian Islands, either arising from a LDD colonization event from a North American ancestor or from an ancestor in Australasia via Rapa Iti (Dunbar‐Co et al., 2008).

Until now, inferences of dispersal patterns within subgenus *Plantago* based on molecular data have been hindered by poorly resolved phylogenetic relationships due to too few of the 130 taxa being available for previous analyses [i.e. Rønsted et al. (2002) included 19 species; Hoggard et al. (2003) included 14 species, Ishikawa et al. (2009) included 24 species; and Tay et al. (2010a) included 20 species] as well as insufficient resolution obtained by the few genetic sequences used. Additionally, only 19 of the 45 island endemics in subgenus *Plantago* were available for previous phylogenetic analyses covering seven islands or island systems with known *Plantago* species (most of these were restricted to Australasia [Meudt, 2012; Tay et al., 2010a], see Appendix I, Supporting Information). This severely limits the testing of biogeographic histories for island taxa in the globally distributed genus *Plantago*.

In this study, molecular data from 14 of the island endemics were obtained – covering 11 of the 18 different oceanic islands or island systems where *Plantago* species are known – and the number of taxa represented from within subgenus *Plantago* was substantially increased (Appendix I, Supporting Information). We include for the first‐time endemic species from Juan Fernandez Islands, Lord Howe Island, Java and Jeju. This sampling increase not only results in improved phylogenetic and biogeographical inference but also allows for a greater number of island calibration points to investigate divergence times within this challenging taxonomic group. We use phylogenetic analyses of nuclear internal transcribed spacer (ITS) sequence data along with sequence data of four plastid regions (*ndhF‐rpl32*,* rpl32‐trnL*,* rps16* and *trnLF*) to produce a well‐supported phylogenetic tree and further aim to (1) resolve the placement of island taxa within subgenus *Plantago*, (2) infer source areas for island taxa by reconstructing the most probable ancestral ranges and resolve competing hypotheses regarding the biogeographic histories of taxa and (3) infer the degree to which biogeographical proximity (nearest landmass) versus extreme long‐distance dispersal events is responsible for the distribution of the taxa in a genus that is likely to be dispersed by birds across extreme long distances.

## 2 MATERIALS AND METHODS

### 2.1 Taxon sampling

The present study follows the most recent and comprehensive circumscription of the genus published by Rahn (1996), except for the Hawaiian taxa, in which case we follow Wagner, Herbst, and Sohmer (1990). To ascertain biogeographic histories of island taxa within subgenus *Plantago*, we sampled as many oceanic island taxa as possible, plus the representation of species from across the global distribution for the subgenus. In total, 11 of the 18 islands or island systems for which species from subgenus *Plantago* are known are represented (Appendix I). In total, 54 taxa were available for our analyses (Table 1), including 48 species belonging to subgenus *Plantago* (representing 37% of the subgenus) of which 14 species (and three varieties) are endemic to oceanic islands. An additional six species from other taxonomic subgenera in *Plantago* was included to confirm monophyly of subgenus *Plantago*. Initially, multiple specimens were obtained for several critical or rarely collected species, including *P. aucklandica, P. canescens* Adams*, P. fernandezia, P. hawaiensis, P. macrocarpa* Cham. & Schltdl.*, P. palmata* Hook.f.*, P. princeps, P. rapensis* and *P. rupicola*, to test DNA amplification on degraded tissue samples. In all of the above cases, the multiple specimens were monophyletic (data not shown), and therefore, only one specimen per species was used in the final analyses, except for the four varieties of the Hawaiian *P. princeps s.l*. (Dunbar‐Co et al., 2008; Wagner et al., 1990). Material from herbarium specimens of *P. robusta* from St. Helena was also obtained and would have provided an additional calibration point, but could not be included due to lack of amplification of the highly degraded DNA.

**Table 1:** List of *Plantago* taxa and outgroups, including specimen information, and DNA regions used in the present study

Rahn No.	Species	Distribution	Voucher information	DNA Bank ID	ITS	*rps16*	*trnLF*	*ndhF*–*rpl32*	*rpl32*–*trnL*
Genus *Plantago* L.									
Subgenus *Plantago*									
Section *Plantago*									
1	*P. canescens* Adams	Northern Asia, northwestern North America	R. Ernst, Alberta, CA, CSU	30434, K	MK487840	MK487941	MK487990	MK487866	MK487899
2	*P. macrocarpa* Cham. & Schltdl.	Northwestern North America*	M. Woodbridge, 181046, OSC, Lincoln, Oregon.	20556, K	EU602337^b^			EU580432^b^	MK487919
3	*P. rupicola* Pilg.	Rapa Iti	T. J. Motley 2678, NY/K, Rapa Iti, Polynesia.	20558, K	EU602338^b^	MK487939	MK487988	EU580427^b^	EU594462^b^
4	*P. princeps* var*. anomala* Rock	Hawaii	Dunbar, 216, PTBG, Hawaii	2297, HPDL	MK487831		MK487983	MK487880	MK487906
4	*P. princeps* var. *laxifolia* A.Gray	Hawaii	Dunbar, 111, PTBG, Maui, Hawaii.	2288, HPDL	EU602322^b^		MK487984	EU580412^b^	EU594447^b^
4	*P. princeps* var. *longibracteata* H.Mann	Hawaii	Dunbar, 287, PTBG, Hawaii	2308, HPDL	MK487832		MK487985	MK487881	MK487909
4	*P. princeps* var*. princeps* Cham. & Schltdl.	Hawaii	Dunbar, 256, PTBG, Hawaii	2303, HPDL	MK487833		MK487986	MK487877	MK487908
5	*P. fernandezia* Bertero ex Barnéoud	Juan Fernández Islands	H. Valdebenito & A. Landero 6595, OS	20546, K	MK487825	MK487938	MK487994	MK487861	MK487930
6	*P. hawaiensis* (A.Gray) Pilg.	Eastern Hawaii	Dunbar, 10, PTBG, Hawaii	1993, HPDL	MK487830		MK487981	MK487876	MK487910
7	*P. pachyphylla* A.Gray	Eastern Hawaii	Dunbar, 59, PTBG, Hawaii	2046, HPDL	MK487829		MK487982	MK487879	MK487907
14	*P. rapensis* Pilg.	Rapa Iti	T. J. Motley 2740, K	20557, K	MK487837	MK487940	MK487987	MK487850	MK487911
15	*P. aucklandica* Hook.f.	Auckland Island	B. D. Rance, No Data	20547, K	MK487823	MK487943	MK487991	MK487854	MK487932
16	*P. fischeri* Engl.	Eastern Africa	Grimshaw, J.M. 93799 K	31944, K	MK487836				MK487900
18	*P. longissima* Decne.	Southern Africa	H. F. Glen 4054, PRE/K, South Africa	20555, K	MK487835	MK487944	MK487968	MK487855	MK487917
20	*P. sparsiflora* Michx.	Southeastern USA	R. LeBlond, 5305, CSU	30433, K	AJ548979c	MK487945	MK487971	MK487883	
21	*P. gentianoides* subsp. *griffithii* (Decne.) Rech.f.	Southern Asia	Koelz, W. 2076	31948, K	MK487839		MK487996		
22	*P. reniformis* Beck	Southeastern Europe	Rønsted 42, C, Cultivated	9446, K	AY101858^a^		AY101914^a^	MK487856	MK487905
23	*P. cornutii* Gouan	Southern Europe	Rønsted 31, C, Cultivated	11180, K	AY101859^a^		AY101915^a^	MK487859	MK487903
24	*P. palmata* Hook.f.	Tropical Africa	Rahn 670, C, Rwanda	9396, K	AY101860^a^	MK487946	AY101916^a^	MK487853	MK487902
25	*P. africana* Verdc.	Eastern Africa	Thulin, M. 1636 K	31942, K	MK487834		MK487970	MK487852	MK487901
26	*P. major* L.	Cosmopolite, Europe and western Asia	Rønsted 41, C, Cultivated	11185, K	AY101861^a^	MK487947	AY101917^a^	MK487860	MK487918
29	*P. asiatica* L.	Southern and eastern Asia	Rønsted 42, C, China	9585, K	AY101862^a^	MK487948	AY101918^a^	MK487874	
30	*P. taquetii* H.Lév.	Korea	H‐K. Choi, 21088,	20551, K	MK487822	MK487949	MK487972	MK487872	MK487923
31	*P. himalaica* Pilg.	Himalaya	Townsend, C.C. 87/159 K	31935, K	MK487827		MK487989	MK487888	MK487926
33	*P*. *erosa* Wall.	India	Luo, Lin‐bo 0148, K	31932, K	MK487821		MK487980	MK487873	
34	*P. incisa* Hassk.	Java	J.L. Filip, H578184‐52, K	11191, K	MK487828				
35	*P. rugelii* Decne.	Eastern North America	Rønsted 37, C, Ontario	9447, K	AY101863^a^	MK487950	AY101919^a^	MK487882	MK487928
36	*P. eriopoda* Torr.	Western North America	P.J. Cotterill, Edmonton, AB	20541, K	MK487824	MK487951	MK487974	MK487889	MK487913
37	*P. tweedyi* A.Gray	USA	Hoggard R, 518, CSU	30436, K	MK487838	MK487952	MK487973	MK487868	MK487912
38	*P. cordata* Lam.	Eastern North America	Allison JR, 12478, CSU	31947, K	MK487841				MK487904
39	*P. hedleyi* Maiden	Lord Howe Island	Crawford 3833, KANU	20549, K	MK487826		MK487995	MK487887	MK487922
40	*P. maxima* Juss. ex. Jacq.	Eastern Europe	Rønsted 28, C, Cutivated	11181, K	AY101864^a^		MK487969	MK487867	MK487897
41	*P. media* L.	Europe	Rønsted 50, C, Cultivated	9441, K	AY101865^a^	MK487942	AY101920^a^	MK487890	MK487898
Section *Micropsyllium* Decne.									
43	*P. tenuiflora* Waldst. & Kit.	Eastern Europe, Central Asia	Rønsted 30, C, Hungary	11186, K	AY101866^a^	MK487953	AY101921^a^	MK487871	MK487895
48	*P. bigelovii* A.Gray	Eastern USA	Sivinski R 5274, CSU	30445, K	MK487819	MK487954	MK487975	MK487870	MK487896
Section *Mesembrynia* Decne.									
52	*P. camtschatica* Link	Eastern Asia	Rahn 684, C	9402, K	MK487842	MK487955	MK487976	MK487875	MK487925
54	*P. depressa* Willd.	Central and eastern Asia	Zhen Yu Li 11339, PE	20544, K	MK487843	MK487956	MK487977	MK487869	MK487929
60	*P. debilis* R.Br.	Australia, Tasmania	Rønsted 45, C, Cultivated	9443, K	AY101868^a^	MK487957	AY101922^a^	MK487885	MK487915
68	*P. raoulii* Decne.	New Zealand	Rahn 692, C, Cultivated	9428, K	AY101867^a^	MK487958	AY101923^a^	MK487884	MK487921
78	*P. stauntonii* Reichardt	New Amsterdam & St. Paul Islands	Rahn 706, C, New Amsterdam & St. Paul Islands	9586, K	AY101870^a^	MK487959	AY101925^a^	MK487886	MK487916
Section *Virginica* Decne. & Steinh. ex Barnéoud									
84	*P. tomentosa* Lam.	South America	Rønsted 29, C, Argentina	11182, K	AY101872^a^	MK487960	AY101927^a^	MK487865	
91	*P. myosuros* Lam.	South America	Rønsted 47, C, Cultivated	9405, K	AY101873^a^	MK487961	AY101928^a^	MK487863	MK487933
93	*P. virginica* L.	Eastern USA	Hoggard R 250, CSU	30428, K	MK487844	MK487962		MK487864	
108	*P. australis* Lam.	North and South America	DTU‐6/94, C, Cultivated	9425, K	AY101874^a^	MK487963	AY101929^a^		
Section *Oliganthos* Barnéoud									
110	*P. barbata* G.Forst.	South America	Hoggard R 528, CSU	30429, K	MK487845	MK487964	MK487992	MK487857	MK487920
111	*P. moorei* Rahn	Falkland Islands	Hoggard R 528, CSU	30431, K	MK487846	MK487965	MK487993	MK487858	MK487931
121	*P. paradoxa* Hook.f.	Tasmania	D. Burns, 2136, 9011116, CBG	30449, K	AJ548969^c^	MK487966	MK487978	MK487862	MK487914
131	*P. muelleri* Pilg.	Australia, Tasmania	Craven 10162, QRS, CSIRO	30437, K	MK487847	MK487967	MK487979		
Subgenus *Bougueria* (Decne.) Rahn									
162	*P. nubicola* (Decne.) Rahn	South America	HHCH 5079, C, Peru.	9639, K	MK487820		AY101948^a^		
Subgenus *Psyllium* (Mill.) Harms & Reiche									
Section *Lanceifolia* Barnéoud									
170	*P. lanceolata* L.	Cosmopolite, Europe and western Asia	Rønsted 33, C, cultivated	9391, K	AY101898^a^	MK487935	AY101952^a^	MK487849	MK487893
205	*P. tandilensis* (Pilg.) Rahn	Eastern Argentina	Rønsted 51, C, Argentina	9488, K	AY101908^a^		AY101961^a^		
Section *Psyllium* (Tourn. ex Juss.) Lam. & DC.									
149	*P. sempervirens* Crantz	Southwestern Europe	Rønsted 27, C, France	9430, K	AY101889^a^	MK487936	AY101942^a^	MK487848	MK487892
Subgenus *Coronopus* (Lam. & DC.) Rahn									
140	*P. coronopus* L.	Mediterranean basin	Rønsted 8, C, Denmark	9439, K	AY101882^a^	MK487937	AY101937^a^	MK487851	MK487894
Genus *Littorella* P.J.Bergius									
145	*L. uniflora* (L.) Asch.	South America	Chase 2798, K, England	2798, K	AY101885^a^	MK487934	AY101940^a^		MK487891

Notes: Herbaria: (C) Natural History Museum of Denmark, University of Copenhagen, Denmark; (CBG) Australian National Botanic Gardens, Canberra, Australia; (CSU) University of Central Oklahoma, USA; (K) Royal Botanic Gardens, Kew, UK; (KANU) University of Kansas, USA; (OS) Ohio State University, USA; (OSC) Oregon State University, USA; (PE) Institute of Botany, Chinese Academy of Sciences, China; (PTBG) National Tropical Botanical Garden, Hawaii, USA; (QRS) SCIRO, Australian National Herbarium, Queensland.

DNA banks: (HPDL) Hawaiian Plant DNA Library; (K) Royal Botanic Gardens, Kew DNA bank.

a Rønsted et al. (2002).

b Dunbar‐Co et al. (2008).

c Hoggard et al. (2003).

* *Plantago macrocarpa* has been reported in northeastern Russia (Hultén, 1930), but no vouchers could be located to confirm this.

Dried leaf material was obtained from the Botanic Gardens of the University of Copenhagen (C), from our in‐house herbarium collection or from collaborators. Genomic DNA samples were obtained from the Hawaiian Plant DNA Library (HPDL) and from new extractions of herbarium collections (K) provided by the DNA bank of the Royal Botanic Gardens, Kew. Origin and voucher information of materials are listed in Table 1. Additionally, 42 sequences from the previous study of Rønsted et al. (2002), 3 from Dunbar‐Co et al. (2008) and 2 from Hoggard et al. (2003) were downloaded from GenBank as listed in Table 1. In total, 17 species were sequenced for the first time in this study, most of the remaining included species were supplemented with additional sequence data, and 176 new sequences were submitted to GenBank.

### 2.2 DNA extractions, amplification and sequencing

Total genomic DNA was extracted from 15 to 30 mg of dried leaf fragments or herbarium material following Rønsted et al. (2002). Amplification of ITS and the *trnLF* intron was performed following Rønsted et al. (2002), while amplification of the intergenic spacers *ndhF*–*rpl32* and *rpl32*–*trnL* followed Dunbar‐Co et al. (2008). The *rps16* intron was amplified following Oxelman, Lidén, and Berglund (1997). Primers used are listed in Appendix II. Amplified products were purified with the Qiagen PCR purification kit (Qiagen, Germany) following the manufacturer's protocols. Cycle sequencing reactions were carried out using the BigDye™ Terminator Mix (Applied Biosystems, USA). Products were run on an ABI 3730 DNA Analyzer according to the manufacturer's protocols (Applied Biosystems, USA) at the Jodrell Laboratory in Kew Gardens, at the National Sequencing Centre, Natural History Museum of Denmark, or by Macrogen Inc. (Europe). Both strands were sequenced for each region for all taxa.

### 2.3 Phylogenetic analysis

Sequences were assembled, edited and subsequently aligned with MAFFT 7.2 (Katoh & Standley, 2013) using the bioinformatics software platform geneious 9.1.8 (www.geneious.com, Kearse et al., 2012). Gaps were coded for all regions following a simple gap coding scheme (Simmons and Ochoterena (2000). The best‐fit nucleotide substitution model for each marker was chosen based on the corrected Akaike information criterion (AICc) as calculated using jmodeltest 2.1.10 (Darriba, Taboada, Doallo, & Posada, 2012). The best‐fit models are listed in Appendix II (Supporting Information). The partitioned data set was analysed with mrbayes 3.2.6 (Ronquist & Huelsenbeck, 2003), running for 5 million generations and sampling every 200 generations. *Littorella uniflora* was set as the outgroup based on the results of previous phylogenetic analyses for *Plantago* (see Hoggard et al., 2003; Rønsted et al., 2002). Chain convergence and ESS parameters were inspected with Tracer 1.6 (Rambaut, Suchard, Xie, & Drummond, 2014) and the first 25% of the trees sampled from the posterior were discarded as burn‐in. A 50% majority rule consensus tree was calculated and visualized together with the posterior probabilities using figtree 1.4.3 (Rambaut, 2012). Maximum likelihood analyses were performed in RaxML (Stamatakis, 2014), defining *L. uniflora* as outgroup and setting the number of bootstrap iterations to 1000.

### 2.4 Divergence time analysis

beast 2.4.7 was used to compute divergence times (Bouckaert et al., 2014). The nucleotide substitution models used in the beast analysis were identical to the ones used in the mrbayes analysis (Appendix II). *Littorella uniflora* was defined as the outgroup. The appropriate molecular clock model was determined by using pathsampler 1.3.4, which is integrated in beast 2.4.7 (Bouckaert et al., 2014). The chain length for this path sampling analysis was set at 1 million generations and the number of steps at 100. The marginal likelihood estimates for a strict, a lognormal and an exponential clock were obtained, and an uncorrelated relaxed lognormal clock was determined to be the most likely model and thus selected for the analyses.

Assuming the timing of dispersal to a datable oceanic island occurred soon after emergence of the island from the ocean, the age of the island can be used as an approximate maximum date for the occurrence of endemic species to that island (Ho et al., 2015; Richardson et al., 2001; Rønsted et al., 2002). In the absence of reliable fossil data for *Plantago*, previous studies of *Plantago* have used the endemicity of *P. stauntonii* on New Amsterdam Island as calibration point (*Plantago* section *Mesembrynia* Decne.; Rønsted et al., 2002; Tay et al., 2010a). In the present study, the ages of five oceanic islands were used as calibration points for the occurrence of endemic species to those islands and a normal distribution was used for the estimation of those priors (Table 2). Using this type of data may cause overestimation of divergence times (Hipsley & Müller, 2014). We therefore applied a large confidence interval for each calibration point, allowing for the lower limit to be zero (i.e. present day). Three independent MCMC runs were performed with an uncorrelated relaxed lognormal clock prior on molecular rates and a restricted calibrated Yule speciation process prior on tree shapes. Chain length for each run was set at 100 million generations and sampling was conducted every 5,000 generations. Chain convergence and ESS parameters were inspected with tracer 1.6 (Rambaut et al., 2014). The burn‐in of each run was removed and the outputs of the three independent runs were pooled using logcombiner 2.4.7 (Bouckaert et al., 2014). A maximum clade credibility tree was produced from the combined file composed of the three replicate runs using treeannotator 2.4.7 (Bouckaert et al., 2014) with mean heights and a posterior probability limit of 0.5 and visualized using figtree 1.4.3 (Rambaut, 2012).

**Table 2:** Oceanic island calibration points used for the divergence time analysis for *subgenus Plantago*

Island	Mean age	Reference	Endemic taxa
Lord Howe Island	6.9 Ma	Zielske, Ponder, & Haase, 2017; McDougall, Embleton, & Stone, 1981	*P. hedleyi*
New Amsterdam Island	0.3 Ma	Doucet, Weis, Scoates, Debaille, & Giret, 2004	*P. stauntonii*
Juan Fernández Islands	5.8 Ma	Anderson, Bernardello, Stuessy, & Crawford, 2001; Stuessy, Foland, Sutter, & Silva, 1984	*P. fernandezia*
Hawaiian Islands	5.1 Ma	Ree & Smith, 2008; Fleischer, McIntosh, & Tarr, 1998	*P. hawaiensis* *P. pachyphylla* *P. princeps s.l*.
Rapa Iti	5.1 Ma	Zielske et al., 2017; Krummenacher & Noetzlin, 1966	*P. rupicola* *P. rapensis*

### 2.5 Biogeographical analysis and ancestral range estimation

The R package ‘biogeoBEARS’ (Matzke, 2013) was used to compare biogeographical models and estimate the ancestral ranges of island taxa in subgenus *Plantago*. The Bayesian maximum clade credibility tree obtained in the beast analysis was used as input. biogeoBEARS uses a maximum likelihood framework (ML) for the three most commonly used models in historical biogeography, the dispersal–extinction–cladogenesis (DEC; Ree & Smith, 2008), dispersal–vicariance analysis (DIVA, Ronquist, 1997) and Bayesian Inference of Historical Biogeography for Discrete Areas (BayArea; Landis, Matzke, Moore, & Huelsenbeck, 2013). The DIVA model and the BayArea model are implemented in ML, rather than the parsimony or Bayesian frameworks originally developed for these models; therefore, the models are called DIVALIKE and BAYAREALIKE in biogeoBEARS (Matzke, 2013). biogeoBEARS also allows for the modelling of founder‐event speciation, with the implementation of the + *j* parameter. We applied seven biogeographical regions in our analyses, following the works of others for plants with global distributions (i.e. Dupin et al., 2017): Europe and western Asia; eastern Asia and Southeast Asia; Africa; Pacific Islands; Australasia; North America; South America. We assigned the area of each species to the seven areas according to distributions listed in Rahn (1996), Flora of China (Missouri Botanical Garden & Harvard University Herbaria, eFloras, 2017) and Flora Iranica (Patzak & Rechinger, 1965). For the cosmopolitan weeds, *P. major* L. and *P. lanceolata*, putative centres of origin were used to code their distribution being from Europe and western Asia (based on the floras listed above). Maximum range areas in biogeoBEARS was set to two areas, based on there being a maximum of two geographic areas occupied by the extant taxa included in our analyses.

All six possible models (DEC, DEC + *j*, DIVALIKE, DIVALIKE + *j*, BAYAREALIKE and BAYAREALIKE + *j*) were fitted to the data, and the selection of the best‐fit model was based on comparing the log‐likelihood values and corrected Akaike Information Criterion (Table 3). The results of the ancestral range analysis are visualized as pie charts on each supported node of the tree, signifying the probabilities for all estimated ranges of all possible biogeographic histories.

**Table 3:** Summary statistics for the six models in BioGeoBEARS for ancestral state reconstruction in *subgenus Plantago*. Abbreviations: log‐likelihood (LnL), dispersal parameter (*d*), extinction parameter (*e*), founder effect parameter (*j*) and corrected Akaike information criterion (AICc). Bold text indicates that the data are best explained by a DEC + j model

Model	LnL	No. Parameters	*d*	*e*	*j*	AICc
DEC	−100.4	2	0.011	8.27E‐3	0	204.7
DEC+J	**−77.6**	**3**	**0.0017**	**1.00E‐12**	**0.032**	**161.3**
DIVALIKE	−99.3	2	0.014	5.4E‐3	0	202.6
DIVALIKE+J	−78.8	3	0.0021	1.00E‐12	0.033	163.5
BAYAREALIKE	−120.3	2	0.019	0.11	0	244.6
BAYAREALIKE+J	−79.5	3	0.0015	1.00E‐07	0.037	164.9

## 3 RESULTS

### 3.1 Phylogenetic relationships and divergence times for section Plantago

**Figure 1:**
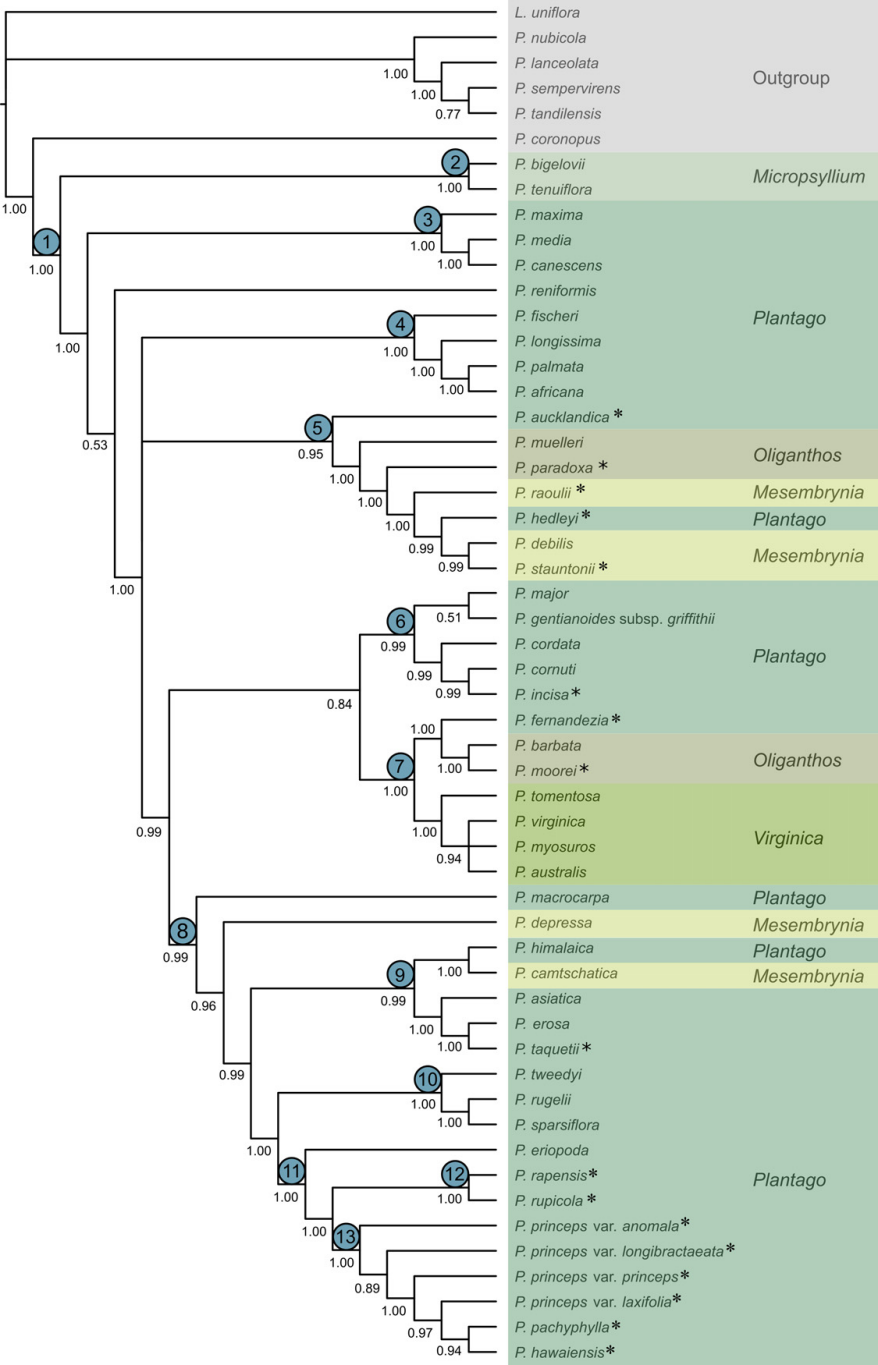
The 50% majority rule consensus cladogram from the mrbayes analysis for members from subgenus *Plantago*. The posterior probabilities are listed below the branches. Taxonomic sections of *Plantago* are shown in different colours. Numbers listed above the nodes are the numbers for clades discussed in the text. Island taxa are denoted by an asterisk (*) following the taxon name [Colour figure can be viewed at wileyonlinelibrary.com]

**Figure 2:**
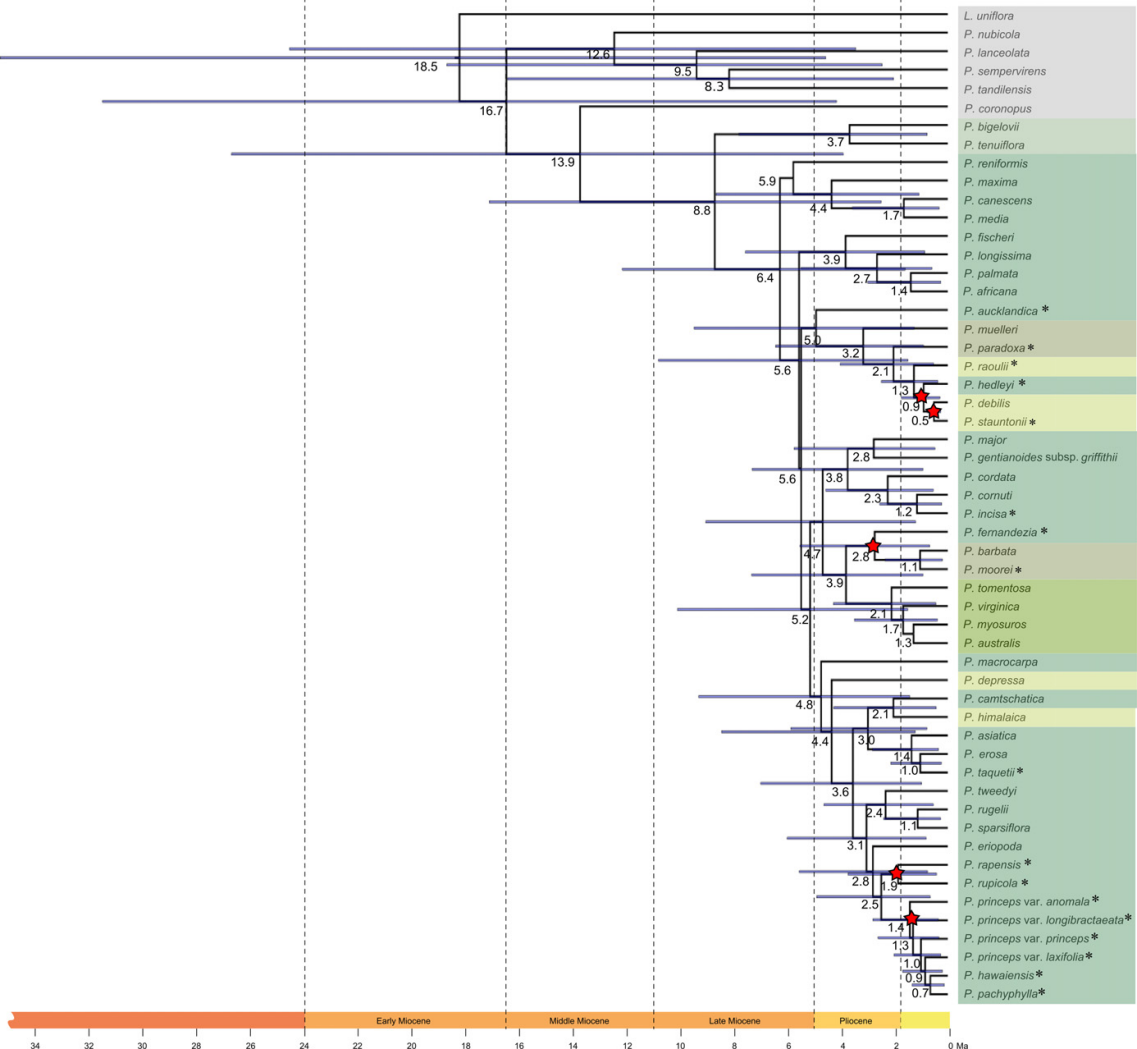
Divergence time tree of subgenus *Plantago* from the beast analysis. The five calibration points are indicated with red stars, and represent (from top to bottom) Lord Howe Island, New Amsterdam Island, the Juan Fernández Islands, Rapa Iti and the Hawaiian Islands. Island taxa from subgenus *Plantago* are denoted by an asterisk (*) following the taxon name [Colour figure can be viewed at wileyonlinelibrary.com]

The resulting alignment from concatenating all sequences from the sampled taxa is 4639 base pairs long; however, not all five regions could be amplified for all taxa (see Table 1). No hard incongruences between the ITS and the plastid topologies were obtained and only the combined tree is presented herein. Separate trees for ITS and plastid regions are presented as supplementary files (Figures S1 and S2), along with a tree from the RAxML analysis (see Supporting Information, Figure S3). Bayesian phylogenetic analysis of the five DNA regions resulted in a robust phylogenetic tree with nodes with high posterior probability (<0.95) in all but a few cases. In the backbone of the topology, the sister relationship of a clade containing *P. canescens, P. maxima* and *P. media* to the remainder of subgenus *Plantago* was only supported by PP = 0.53, and a trichotomy was found consisting of a European clade (3), a Hawaiian clade (13) and the remainder of subgenus *Plantago*. The consensus tree and posterior probabilities (PP) from the mrbayes analysis are presented in Figure 1, and the results of the beast divergence analyses are presented in Figure 2. Island endemics are indicated with an asterisk (“*”) in both Figures 1 and 2. Current geographic distribution for all taxa is listed in Table 1. Maximum likelihood analysis resulted in the same topology, though support was lower at many nodes (Supplementary Information, Figure S3).

Subgenus *Plantago* is found to be monophyletic (Clade 1; PP = 1.00; Figure 1) and started diverging 8.8 Ma (with an error range of 17.4–2.5 Ma) (Figure 2). The 14 sampled island taxa were resolved in six different clades (Clades 5, 6, 7, 9, 12, 13) and each is described below. All divergence time estimates presented in the text are followed by error estimates in square parentheses based on the HPD 95% confidence intervals (as shown in Figure 2).

Two taxa classed into section *Micropsyllium* Decne. form a monophyletic clade (Clade 2; PP = 1.00; 3.9 Ma (7.9–0.8 Ma) which is sister to the rest of the subgenus. The other three taxonomic sections (as defined by Rahn, 1996) within the subgenus are polyphyletic. There are up to 12 different clades that can be recognized within subgenus *Plantago*, all of which have high support. These clades are mainly formed by species that share common geographic distributions rather than taxonomic sections as previously described (Rahn, 1996). Of particular note are the oceanic island species *P. aucklandica*,* P. hedleyi* and *P. fernandezia*, which are taxonomically classed as part of the section *Plantago* but are phylogenetically clustered with species from other sections in clades that constitute coherent geographic groups.

**Figure 3:**
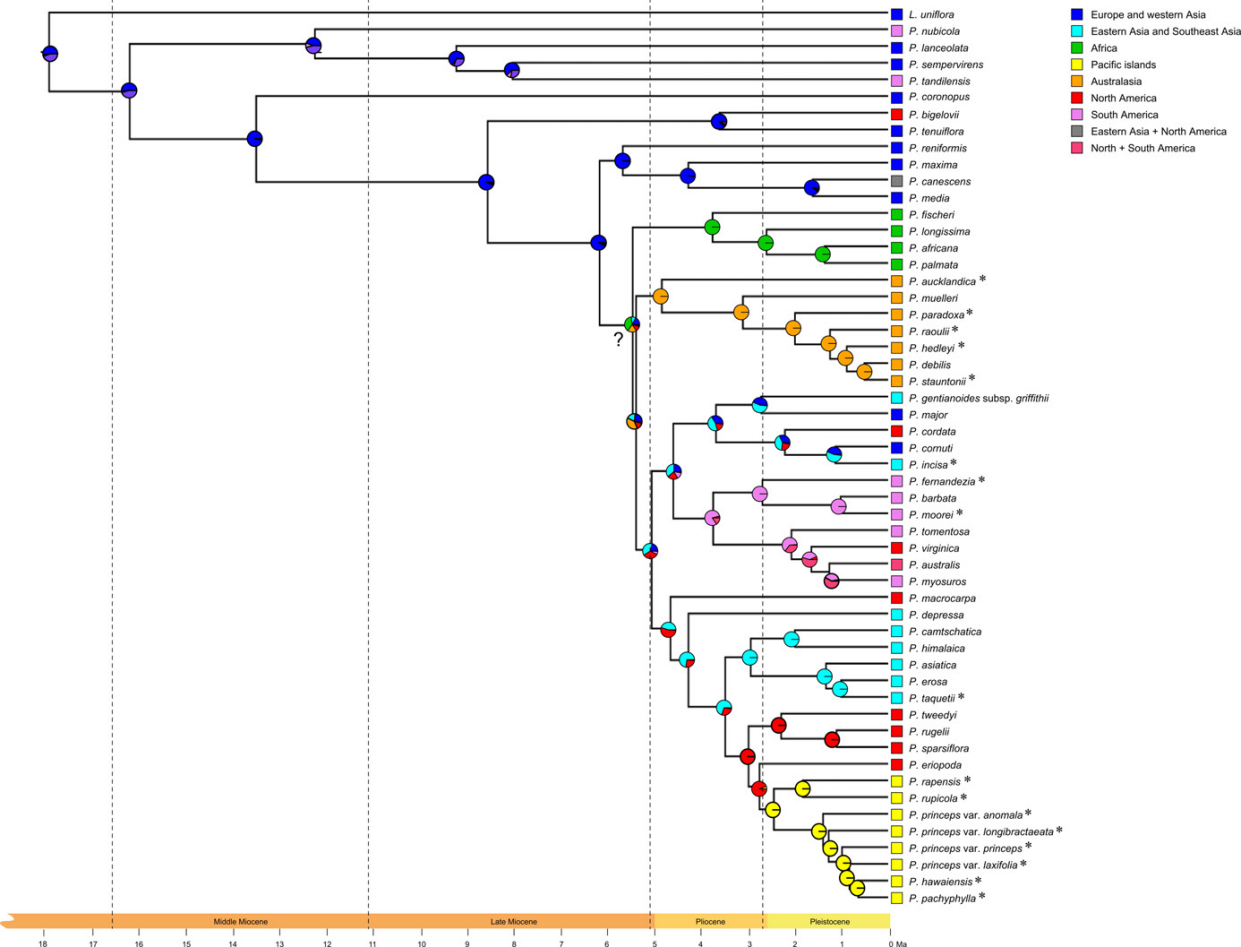
Ancestral range reconstruction of subgenus *Plantago* (−LnL = 76.98) under the best‐fitted model, DEC + *j*, based on the divergence time tree from the beast analysis and the distribution ranges of the extant species. The relative probabilities of the estimated ranges are represented in pie charts at each supported node. Colours correspond to the coded geographic states (single and two area ranges) listed in the legend. The node labelled with a question mark “?” denotes doubtful results from the biogeographical analyses based on poor node support in the mrbayes analyses. Island taxa from subgenus *Plantago* are denoted by an asterisk (*) following the taxon name [Colour figure can be viewed at wileyonlinelibrary.com]

A clade of European species is found to be the next diverging lineage (Clade 3; PP = 1.00; 6.0 Ma [8.8–1.1 Ma]) and consists of the species *P. media* L., *P. maxima* Juss. ex. Jacq. and *P. canescens*, though the last species also occurs in North America (Table 1). A minor incongruence between the mrbayes and beast trees is the positioning of the European species *P. reniformis* (compare Figures 2 and 3). In the beast analysis (Figure 2), *P. reniformis* is placed closest to the European clade, but the relationship between them is uncertain due to low node support. However, in the mrbayes analyses (Figure 1), *P. reniformis* is closer to the rest of the subgenus than to the three species in Clade 3, though also with low support (PP = 0.53).

A clade of African species (all previously classed in section *Plantago*) is also found (Clade 4; PP = 1.00; 4.0 Ma (7.7–0.88 Ma); *P. africana* Verdc., *P. fischeri* Engl., *P. longissima* Decne. and *P. palmata*).

Clade 5 is a highly supported group of Australasian taxa (PP = 0.94; 5.1 Ma) that consists of a mix of members of sections *Oliganthos* Barnéoud and *Mesembrynia*, and includes the largest number of island endemics including *P. aucklandica* from the Auckland Islands, *P. paradoxa* Hook.f. from Tasmania, *P. raoulii* from New Zealand, *P. stauntonii* from Amsterdam Island and *P. hedleyi* from Lord Howe Island. Within this Australasian clade, *P. aucklandica* is found to diverge the earliest of all island taxa sampled in the subgenus (5.1 Ma [9.6–1.3 Ma]), whereas *P. stauntonii* from New Amsterdam Island is found to be the most recently diverged oceanic island endemic in this clade (0.5 Ma [0.8–0.3 Ma]).

Clade 6 consists of a mix species that occur in various geographic areas (PP = 0.99; 3.8 Ma [7.4–0.9 Ma]): the cosmopolitan *P. major, P. gentianoides* subsp. *griffithii* (Decne.) Rech.f. from western Asia, *P. cordata* Lam. from North America, *P. cornutii* Gouan from Europe and western Asia and the island species *P. incisa* Hassk. from Java.

Clade 7 (PP = 1.00; 3.9 Ma [7.4–0.9 Ma]) is an entirely South American clade comprised of taxa classed in section *Virginica* Decne. & Steinh. ex Barnéoud (*P. australis* Lam., *P. myosuros* Lam., *P. tomentosa* Lam. and *P. virginica* L.), and South American taxa from section *Oliganthos*, including the single island endemics *P. fernandezia*, which diverged 2.8 Ma [5.6–0.7 Ma] on the Juan Fernández Islands, and *P. moorei* Rahn from the Falkland Islands which diverged 1.1 Ma [2.4–0.2 Ma]).

A large and more recently diverged clade (Clade 8; PP = 0.99; 4.8 Ma [9.4–1.4]) includes a mixture of taxa from North America, eastern Asia and species from Pacific Island systems, Rapa Iti and Hawaii.

Clade 9 (PP = 0.99; 3.0 Ma [5.9–0.8 Ma]) consists of the majority of the eastern Asian species from sections *Plantago* (*P. asiatica*,* P. erosa* Wall. and *P. himalaica* Pilg.) and *Mesembrynia* (*P. camtschatica* Link), including *P. taquetii* H.Lév., from Jeju which diverged (1.0 Ma [2.2–0–2 Ma].

Clade 10 (PP = 1.00; 2.4 Ma) is a North American group containing the species *P. rugelii* Decne., *P. sparsiflora* Michx. and *P. tweedyi* A.Gray.

In Clade 11 (P = 1.00; 2.8 Ma [5.6–0.8 Ma]), *P. eriopoda* Torr. from western North America is sister to the two Rapa Iti taxa (Clade 12; PP = 1.00; 1.9 Ma [3.8–0.4 Ma]; *P. rapensis* and *P. rupicola*) and the Hawaiian Islands taxa (Clade 13; PP = 1.00; 1.5 Ma [2.8–0–4]; *P. princeps s.l*., *P. hawaiensis* and *P. pachyphylla*).

### 3.2 Ancestral range reconstruction

Model selection for the ancestral range reconstruction indicated that the data are best explained by a DEC + *j* model (LnL = −76.98 and AICc = 160.4; Table 3). The results of the ancestral range reconstruction are depicted in Figure 3, where the probabilities for possible ancestral areas are shown in pie charts at each supported node. The selection of the DEC + *j* model indicates that founder‐event speciation (i.e. the parameter *j*) is a major contributor to the currently observed biogeographical patterns within section *Plantago*.

Interpreting the results of the ancestral range reconstruction at the deeper nodes should be done carefully as incomplete taxon sampling has a major influence on the outcome, and in particular, the presence of phylogenetic uncertainty at some nodes may hinder a correct interpretation. For example, the result presented at the node directly above an unsupported node (indicated with a question mark “?” in Figure 3) is doubtful. The most recent common ancestor (MRCA) of the African clade (Clade 4), the Australasian clade (Clade 5) and the remainder of the tree cannot be confidently inferred, as long as the phylogenetic relationships between these groups are not completely resolved. However, the ancestral ranges at other nodes that are well supported can be estimated with higher confidence.

Based on our sampling, ancestral range reconstruction shows that subgenus *Plantago*, as well as the first diverging clades, likely has geographic origins in Europe. The MRCA of the African species in section *Plantago* is African and the MRCA of the Australasian species, including the island endemics from Lord Howe Island, the Auckland Islands, New Zealand, Tasmania and Amsterdam Island originated in Australasia. The ancestral range of the MRCA of the remaining species is inferred to be in the northern hemisphere, likely in eastern Asia or North America. From there, some taxa could have recolonized Europe or may be descendants of early European ancestors (*P. major* and *P. cornutii*), while other taxa moved into South America, finally also giving rise to the island endemics from the Juan Fernández Islands (*P. fernandezia*) and Falkland Islands (*P. moorei*). The MRCA of *P. macrocarpa* and the remainder of the species are indicated as North American because *P. macrocarpa* was coded as North American in our analysis. However, if unconfirmed reports of *P. macrocarpa* being present in Asia are correct (Hultén, 1930), there would be a greater likelihood that the ancestor is Asian. The ancestral ranges reconstructed at the more recent nodes are more straightforward and the analysis shows that the island endemics from Rapa Iti and the Hawaiian Islands have their most likely ancestral origins in North America, meanwhile *P. taquetii* from Jeju is inferred to have origins in Asia.

## 4 DISCUSSION

### 4.1 Phylogenetic relationships and divergence times

The current work is the most comprehensive phylogenetic and biogeographical study for subgenus *Plantago* to date – and the first to focus on estimating ancestral origins of island endemics. The most important phylogenetic result of this study was that taxa within the subgenus, particularly from sections *Mesembrynia*,* Oliganthos* and *Plantago*, form clades based on their geographic proximities, rather than on previous taxonomic placement. Phylogenetic relatedness, based on our molecular data, is thus found to be incongruent with morphological phylogenetic analyses and previous taxonomic classifications (i.e. Rahn, 1996). Thus, morphological traits are not always useful in inferring evolutionary relationships for this group. Rahn (1996) admitted that taxonomic sections such as section *Plantago* that are geographically inconsistent were evidently paraphyletic in relation to the rest of subgenus *Plantago*, and he urged for further studies to be done to clarify the taxonomy and phylogeny of the species included in this group. With our expanded molecular data set, we confirm the earlier findings of Rønsted et al. (2002), Hoggard et al. (2003), Ishikawa et al. (2009) and Tay et al. (2010a) that sections within subgenus *Plantago* are polyphyletic, and echo that the concepts of taxonomic sections in subgenus *Plantago* need to be revisited.

Using five islands as calibration points, we obtained a date of 8.8 Ma [17.4–2.5 Ma] for the diversification of subgenus *Plantago*. As a consequence, our estimate of the origin and diversification of the entire genus is older than previously estimated, however within the error margins since earlier works dated the split of *Plantago‐Littorella* from *Aragoa* at 7.1 or 2.8 Ma (Rønsted et al., 2002; Tay et al., 2010a). The dating approach used by Tay et al. (2010a) was similar to the approach used herein (using a Bayesian modelling in beast), but only a single calibration point was used (i.e. the young New Amsterdam Island) and therefore recovered a much earlier date of 2.8 Ma (Tay et al., 2010a). Known records for fossil pollen for the genus only extend to the Late Miocene at approximately 6 Ma (Mueller, 1981; Rahn, 1996; Rønsted et al., 2002), though it is possible that the genus is older because fossil ages define only minimum ages. Our divergence analyses were limited to using beast, which in comparative dating analyses have previously been shown to give older estimates of dates compared to other methods (Goodall‐Copestake, Harris, & Hollingsworth, 2009). Regardless of the timing of the origin of subgenus *Plantago*, our analyses are still congruent with earlier hypotheses of recent speciation (Rønsted et al., 2002; Tay et al., 2010a), especially in lineages with island endemics, such as the Hawaiian Islands, New Amsterdam Island and Lord Howe that are inferred to have diverged most recently in the group, sometime in the Pleistocene.

Three of the island taxa were resolved within clades different from their classification in taxonomic sections by Rahn (1996; *P. aucklandica* and *P. hedleyi* were resolved within Australasian sections *Oliganthos* and *Mesembrynia*; and *P. fernandezia* in a clade with South American sections *Oliganthos* and *Virginica*). Interestingly, *P. aucklandica* was found to diverge much earlier than any of the other island taxa in our study, which is in keeping with the findings of Tay et al. (2010a) that *P. aucklandica* is part of an early diverging clade, which was sister to all other Australasian taxa in subgenus *Plantago*. Considering that less than half of all known species described in subgenus *Plantago* were included in the current work, further investigation should focus on a more comprehensive sampling of species from the subgenus to resolve these relationships, taxonomy and divergence times with higher certainty.

### 4.2 Historical biogeography of island endemics

Our sampling in subgenus *Plantago* was extensive enough to infer a first approximation of the biogeographical history for subgenus *Plantago* and suggest ancestral ranges of the 14 island taxa we sampled. The ancestor to all subgenus *Plantago* likely originated in Europe. However, the island endemics were found to come from six different lineages each with different ancestral ranges. This suggests that several lineages within subgenus *Plantago* were successful in dispersing to, and speciating on, oceanic islands, which is not surprising for a group with a global distribution and adaptations to bird dispersal (Birch & Keeley, 2013; de Queiroz, 2005). It confirms earlier theories that the genus *Plantago* is particularly efficient at dispersing, especially over long ranges (Rønsted et al., 2002; Tay et al., 2010a).

Our analyses further show that different patterns of dispersal are inferred for island taxa in *Plantago*. Geographic proximity is found to be a key factor in determining relatedness and defining the biogeographic histories of some of the island taxa within subgenus *Plantago*, such that the nearest continents and landmasses were inferred to be source areas for the taxa endemic to Lord Howe Island, Auckland Islands, New Zealand, Tasmania, Juan Fernández Islands and the Falkland Islands. Only in the case of the most remote islands in our study, that is, the Hawaiian Islands and Rapa Iti, and New Amsterdam Island, do we find evidence of extreme long‐distance events that defy the rules of proximity, which is in keeping with previous findings on the origins of island floras such as Hawaii (Baldwin & Wagner, 2010). Interestingly, it is only on these remote islands that multiple *Plantago* species are known; the remaining islands host single island endemics. The specific biogeographical findings for each island taxon included in this study are discussed below (and illustrated in Figures 1, 2, 3.

*Plantago fernandezia,* from the Juan Fernández Islands off the coast of Chile, and *P. moorei* from the Falkland Islands were inferred to have been dispersed from ancestors of close geographic proximity in South America. *Plantago fernandezia* was previously thought to be more closely related to other island taxa from Hawaii and Rapa Iti because it shares more morphological features (such as woody stems typical of insular taxa) in common with them (Pilger, 1937; Rahn, 1996). Our results are, however, in line with the biogeographical histories of other plant lineages from the Juan Fernández Islands having their sources in neighbouring South America (Stuessy et al., 2018). This result also supports the idea that, at least in the case of island taxa, growth forms such as woodiness and other morphological traits (i.e. the presence of only two ovules) can be derived rather than of relict origin and reflect convergent evolution (Carlquist, 1970; Emerson, 2002) rather than phylogenetic relationships.

*Plantago hedleyi* from Lord Howe Island, *P. aucklandica* from the Auckland Islands, the mainland New Zealand taxon (*P raoulii*) and Tasmanian species (*P. paradoxa*) have their ancestral ranges in Australasia (Figure 3). *Plantago stauntonii* from New Amsterdam Island was also found to have ancestral ranges in Australasia, despite the island being situated midway between Africa and Australia, and that representatives of extant species in subgenus *Plantago* are known from both continents. Molecular phylogenetic studies aimed at reconstructing origins of New Amsterdam flora are scarce; however, Bauret et al. (2017) hypothesized that a likely dispersal scenario for a fern species endemic to New Amsterdam was from Madagascar via the Neotropics. We demonstrate that Australasia is an important floristic source for *Plantago* on Amsterdam Island.

*Plantago incisa* from Java has its ancestral range within Europe or Asia though it is phylogenetically most closely related to European species, and is geographically closer to the Australasian species. Sampling other *Plantago* species from New Guinea and mainland Asia would be needed to improve confidence in our ancestral range estimations.

Island taxa from the Hawaiian Islands and Rapa Iti were found to have a common ancestor in western North America, thousands of kilometres further away from their closest landmasses. *Plantago* species from the Hawaiian Islands have previously been proposed to have arisen from a single LDD colonization event either from a North American ancestor or from an ancestor in New Zealand via Rapa Iti (Dunbar‐Co et al., 2008). Our findings rule out the possibility of Hawaiian *Plantago* originating from New Zealand and dispersing via a stepping stone pattern, despite evidence of dispersal moving in that direction for some Hawaiian plant groups (Dunbar‐Co et al., 2008; and references therein; Birch & Keeley, 2013). North America is increasingly recognized as the source for the majority of Hawaiian lineages due the Pacific flyway (Baldwin & Wagner, 2010). Despite the seemingly higher possibility of stepping stone dispersal or island hoping driving the dispersal of island taxa in the Pacific, extreme long‐distance dispersal events are now considered equally as likely and explain the biogeographies of many globally distributed plant groups (Birch & Keeley, 2013; Dupin et al., 2017; Gillespie et al., 2012; Givnish et al., 2009; Nathan, 2006). Our findings therefore further demonstrate the importance of the Pacific flyway and the occurrence of extreme LDD events for the movement of flora from North America across thousands of kilometres to the Hawaiian Islands and also Rapa Iti (Baldwin & Wagner, 2010). Given that the Hawaiian Islands and Rapa Iti have multiple endemic *Plantago* species present on them and are among the most extreme with regard to island remoteness in our study group, these island areas may represent the most extreme cases of recent speciation in island *Plantago* lineages. Further genetic and ecological study of these species may assist in determining what traits are important in not only successful dispersal and colonization to islands but also the subsequent diversification (Baker, 1955, 1955; Carvajal‐Endara, Hendry, Emery, & Davies, 2017).

Although our analyses were sufficient to provide a first approximation of ancestral ranges of island taxa, ancestral range reconstruction at one of the deeper nodes in the tree (shown with a question mark “?” in Figure 3) is highly uncertain due to poorly supported phylogenetic relationships, and increased sampling from subgenus *Plantago* would be necessary to improve node support and confidence in the ancestral range reconstruction, and clarify the biogeographic histories and relationships between extant *Plantago* taxa from Africa, Asia and Australasia. For example, our sampling and/or the molecular data produced herein was insufficient to test whether dispersal to Australasia came via the west (Africa) or north (Asia). A more comprehensive sampling of subgenus *Plantago* would be needed in order to further emphasize that, for biogeographic studies such as this, it is the phylogenetic sampling that is critical, not the taxonomy as proposed by Rahn (1996). Additionally, the definition of biogeographic regions used in our analyses limits the testing of ancestry of insular taxa being in temperate locations (van der Aart & Vulto, 1992). Future analyses could investigate the degree of climate niche matching between island taxa and their ancestral ranges.

### 4.3 Long‐distance dispersal by birds

Our findings support the notion that extant plant species in subgenus *Plantago* are well adapted to dispersal (Rønsted et al., 2002; Tay et al., 2010a), and this was also likely the case for common ancestors. However, the distance that the propagules can disperse may be a factor of the type of birds involved rather than differences in propagule traits. Due to the closer proximity between some oceanic islands to their nearest landmasses (i.e. Lord Howe 600 km from Australia, Auckland Island 500 km south of New Zealand, Juan Fernández 600 km off the coast of Chile and the Falkland Islands 480 km off the coast of Argentina), the dispersal patterns we inferred may be due to movement by smaller birds (Nogales, Heleno, Traveset, & Vargas, 2012; and references therein). Meanwhile, the extreme long‐distance dispersal patterns inferred for taxa on the Hawaiian Islands, as well as on Rapa Iti, coincide with the migration of large marine birds, such as the Pacific Plover, that fly thousands of kilometres from the coasts of Alaska to the Pacific Islands over the Pacific flyway (Gillespie et al., 2012; Henshaw, 1910; Jenni & Jenni‐Eiermann, 1998; Nogales et al., 2012). Similarly, *Plantago* may have found its way to New Amsterdam Island (midway between Australia and Africa in the southern Indian Ocean) with the help of larger sea birds or accidental birds capable of flying thousands of kilometres. However, the rarity of LDD events and limitations in studying historical bird movements and behaviour makes it difficult to conclude which birds may have been responsible (Nogales et al., 2012).

Our findings show that, for a globally distributed plant genus that is well suited to LDD by birds, differing scales and modes of dispersal may be equally important in explaining the biogeographical histories. Similarly, differing dispersal modes have also been inferred in explaining the historical biogeographies of other bird‐dispersed plant families with unique taxa on multiple oceanic islands (i.e. Asteliaceae [Birch & Keeley, 2013], Rubiaceae [Kainulainen et al., 2017]), and thus, evidence is building that there is not a single LDD model that fits all, but rather that a combination of stepping stone dispersal and extreme LDD can both shape insular floras within closely related plant groups, and that multiple floristic areas can be the sources of closely related island taxa.

### 4.4 Limitations to diversification

*Plantago* is an example of a plant group, which is particularly well adapted to long‐distance dispersal, and possesses traits such as wind pollination and self‐compatibility as well as ability to grow in harsh environments that are conducive to establishment and speciation in insular settings (Baker, 1955). However, the majority of *Plantago* taxa are single island endemics and thus not as successful in radiation and speciation in the insular setting compared to, for example, ferns that are generally over‐represented on islands (Hennequin, Kessler, Lindsay, & Schneider, 2014). As plants with adaptations to LDD such as *Plantago* are not always over‐represented in island floras, it is increasingly being accepted that factors other than dispersal limitations are important for the successful colonization and speciation of insular species (Baker, 1955; Carvajal‐Endara et al., 2017; Cheptou, 2012; Heleno & Vargas, 2015). For example, the flora of the Galápagos Islands – of which, 30% are single island endemics – was found to be shaped by habitat filtering rather than by dispersal limitations. Consequently, the match between the species continental climate niche and the island climate was the single best predictor of colonization success (Carvajal‐Endara et al., 2017). In the case of orchids, specific traits such as their pollination biology and association with mycorrhizal fungi are considered factors limiting successful colonization (McCormick & Jacquemyn, 2014). The genus *Plantago* thus remains an interesting case not only to study long‐distance dispersal patterns but also to test hypothesis for limitations to successful establishment and radiation in insular habitats.

## 5 CONCLUSIONS

Our study provides further insights into the importance of geographic proximity as sources of island flora, even for a group of plants that is arguably well adapted to long‐distance dispersal by birds, and that differing scales and modes of dispersal may be equally important in explaining the biogeographical histories of insular species. This further suggests that factors other than dispersal success are important for the establishment and subsequent speciation of insular taxa. This work further emphasizes that classical cladistics approaches to infer closely related species (i.e. using morphological traits) can often mislead the reconstruction of accurate biogeographical histories of island species (de Queiroz, 2005); however, using molecular data to infer ancestral ranges can greatly improve our understanding of biogeographical histories and help elucidate origins, dispersal routes and means in widespread lineages with complex distribution patterns such as *Plantago*. The genus *Plantago* is an excellent case to study to further improve our understanding of organismal traits and ecological factors involved in the successful colonization of insular habitats.

## BIOSKETCH

Natalie Iwanycki Ahlstrand's research interests are in using multidisciplinary techniques to retrace migration and dispersal patterns of useful plants, in evolutionary time‐scales and also in the Anthropocene. This work was a part of her PhD research on the dispersal, migration and local adaptation of species with global distributions in the genus *Plantago* (see http://snm.ku.dk/da/plant_evolutionary_interactions/).

Author contributions: N.R., N.I.A. and R.H. conceptualized the study and assembled the plant material. N.R., R.H. and S.D.C. performed DNA extractions, and NR and RH conducted DNA sequencing. N.I.A. and B.V. processed and analysed sequence data. N.I.A. wrote the manuscript with B.V. and N.R. All authors read and commented on the manuscript and approved the final version. Authors declare no conflicts of interest.

## Data Availability

All newly generated sequence data for this study have been submitted to GenBank.

## References

[jbi13525-bib-0001] Anderson, G. J. , Bernardello, G. , Stuessy, T. F. , & Crawford, D. J. (2001). Breeding system and pollination of selected plants endemic to Juan Fernández Islands. American Journal of Botany, 88, 220–233. 10.2307/2657013 11222245

[jbi13525-bib-0002] Bacon, C. D. , Simmons, M. P. , Archer, R. H. , Zhao, L. C. , & Andriantiana, J. (2016). Biogeography of the Malagasy Celastraceae: Multiple independent origins followed by widespread dispersal of genera from Madagascar. Molecular Phylogenetics and Evolution, 94, 365–382. 10.1016/j.ympev.2015.09.013 26432393

[jbi13525-bib-0003] Baker, H. G. (1955). Self‐compatibility and establishment after ‘long distance’ dispersal. Evolution, 9, 347–348.

[jbi13525-bib-0004] Baldwin, B. G. , & Wagner, W. L. (2010). Hawaiian angiosperm radiations of North American origin. Annals of Botany, 105, 849–879. 10.1093/aob/mcq052 20382966 PMC2876002

[jbi13525-bib-0100] Bauret, L. , Rouhan, G. , Hirai, R. Y. , Perrie, L. , Prado, J. , Salino, A. , & Gaudeul, M. (2017). Molecular data, based on an exhaustive species sampling of the fern genus Rumohra (Dryopteridaceae), reveal a biogeographical history mostly shaped by dispersal and several cryptic species in the widely distributed Rumohra adiantiformis. Botanical Journal of the Linnean Society, 185, 463–481.

[jbi13525-bib-0005] Bello, M. A. , Chase, M. W. , Olmstead, R. G. , Rønsted, N. , & Albach, D. (2002). The páramo endemic *Aragoa* is the sister genus of *Plantago* (Plantaginaceae; Lamiales): Evidence from plastid *rbcL* and nuclear ribosomal ITS sequence data. Kew Bulletin, 57, 585–597. 10.2307/4110987

[jbi13525-bib-0006] Birch, J. L. , & Keeley, S. C. (2013). Dispersal pathways across the Pacific: The historical biogeography of *Astelia* s.l. (Asteliaceae, Asparagales). Journal of Biogeography, 40, 1914–1927.

[jbi13525-bib-0007] Bouckaert, R. , Heled, J. , Kuhnert, D. , Vaughan, T. , Wu, C. H. , Xie, D. , … Drummond, A. J. (2014). beast 2: A software platform for Bayesian evolutionary analysis. PLoS Computational Biology, 10, e1003537 10.1371/journal.pcbi.1003537 24722319 PMC3985171

[jbi13525-bib-0008] Buse, T. , & Filser, J. (2014). Mucilaginous seeds and algal diets attract soil *Collembola* in preference tests. European Journal of Soil Biology, 65, 706–6. 10.1016/j.ejsobi.2014.08.005

[jbi13525-bib-0009] Carlquist, S. (1966). The biota of long‐distance dispersal. I. Principles of dispersal and evolution. The Quarterly Review of Biology, 41, 247–270. 10.1086/405054 5975995

[jbi13525-bib-0010] Carlquist, S. (1967). The biota of long‐distance dispersal. V. Plant dispersal to Pacific Islands. Bulletin of the Torrey Botanical Club, 94, 129–162. 10.2307/2484044

[jbi13525-bib-0011] Carlquist, S. (1970). Wood anatomy of insular species of *Plantago* and the problem of raylessness. Bulletin of the Torrey Botanical Club, 97, 353–361. 10.2307/2483855

[jbi13525-bib-0012] Carvajal‐Endara, S. , Hendry, A. P. , Emery, N. C. , & Davies, J. T. (2017). Habitat filtering not dispersal limitation shapes oceanic island floras: Species assembly of the Galápagos archipelago. Ecology Letters, 20, 495–504. 10.1111/ele.12753 28294532

[jbi13525-bib-0013] Cheptou, P.‐O. (2012). Clarifying Baker's Law. Annals of Botany, 109, 633–641. 10.1093/aob/mcr127 21685434 PMC3278284

[jbi13525-bib-0014] Christenhusz, M. J. M. , & Chase, M. W. (2013). Biogeographical patterns of plants in the Neotropics—Dispersal rather than plate tectonics is most explanatory. Botanical Journal of the Linnean Society, 171, 277–286. 10.1111/j.1095-8339.2012.01301.x

[jbi13525-bib-0015] Czarnecka, J. , & Kitowski, I. (2013). The white stork as an engineering species and seed dispersal vector when nesting in Poland. Annales Botanici Fennici, 50, 706–12. 10.5735/085.050.0101

[jbi13525-bib-0016] Darriba, D. , Taboada, G. L. , Doallo, R. , & Posada, D. (2012). jModelTest 2: More models, new heuristics and parallel computing. Nature Methods, 9, 772–772. 10.1038/nmeth.2109 PMC4594756 22847109

[jbi13525-bib-0017] de Queiroz, A. (2005). The resurrection of oceanic dispersal in historical biogeography. Trends in Ecology & Evolution, 20, 68–73. 10.1016/j.tree.2004.11.006 16701345

[jbi13525-bib-0018] Doucet, S. , Weis, D. , Scoates, J. S. , Debaille, V. , & Giret, A. (2004). Geochemical and Hf‐Pb‐Sr‐Nd isotopic constraints on the origin of the Amsterdam‐St. Paul (Indian Ocean) hotspot basalts. Earth and Planetary Science Letters, 218, 179–195. 10.1016/S0012-821X(03)00636-8

[jbi13525-bib-0019] Dunbar‐Co, S. , Sporck, M. J. , & Sack, L. (2009). Leaf trait diversification and design in seven rare taxa of the Hawaiian *Plantago* radiation. International Journal of Plant Science, 170, 61–75. 10.1086/593111

[jbi13525-bib-0020] Dunbar‐Co, S. , Wieczorek, A. M. , & Morden, C. W. (2008). Molecular phylogeny and adaptive radiation of the endemic Hawaiian *Plantago* species (Plantaginaceae). American Journal of Botany, 95, 1177–1188. 10.3732/ajb.0800132 21632435

[jbi13525-bib-0021] Dupin, J. , Matzke, N. J. , Sarkinen, T. , Knapp, S. , Olmstead, R. G. , Bohs, L. , & Smith, S. D. (2017). Bayesian estimation of the global biogeographical history of the Solanaceae. Journal of Biogeography, 44, 887–899. 10.1111/jbi.12898

[jbi13525-bib-0022] Emerson, B. C. (2002). Evolution on oceanic islands: Molecular phylogenetic approaches to understanding pattern and process. Molecular Ecology, 11, 951–966. 10.1046/j.1365-294X.2002.01507.x 12030975

[jbi13525-bib-0023] Fischer, M. H. , Yu, N. , Gray, G. R. , Ralph, J. , Anderson, L. , & Marlett, J. A. (2004). The gel‐forming polysaccharide of psyllium husk (*Plantago ovata* Forsk). Carbohydrate Research, 339, 2009–2017. 10.1016/j.carres.2004.05.023 15261594

[jbi13525-bib-0024] Fleischer, R. C. , McIntosh, C. E. , & Tarr, C. L. (1998). Evolution on a volcanic conveyor belt: Using phylogeographic reconstructions and K‐Ar‐based ages of the Hawaiian Islands to estimate molecular evolutionary rates. Molecular Ecology, 7, 533–545. 10.1046/j.1365-294x.1998.00364.x 9628004

[jbi13525-bib-0025] Gallaher, T. , Callmander, M. W. , Buerki, S. , & Keeley, S. C. (2015). A long‐distance dispersal hypothesis for the Pandanaceae and the origins of the *Pandanus tectorius* complex. Molecular Phylogenetics and Evolution, 83, 20–32. 10.1016/j.ympev.2014.11.002 25463018

[jbi13525-bib-0026] Gillespie, R. G. , Baldwin, B. G. , Waters, J. M. , Fraser, C. I. , Nikula, R. , & Roderick, G. K. (2012). Long‐distance dispersal: A framework for hypothesis testing. Trends in Ecology and Evolution, 27, 47–56. 10.1016/j.tree.2011.08.009 22014977

[jbi13525-bib-0027] Givnish, T. J. , Millam, K. C. , Evans, T. M. , Hall, J. C. , Pires, J. C. , Berry, P. E. , & Sytsma, K. J. (2004). Ancient vicariance or recent long‐distance dispersal? Inferences about phylogeny and South American‐African disjunctions in Rapateaceae and Bromeliaceae based on *ndhF* sequence data. International Journal of Plant Sciences, 165, S35–S54. 10.1086/421067

[jbi13525-bib-0028] Givnish, T. J. , Millam, K. C. , Mast, A. R. , Paterson, T. B. , Theim, T. J. , Hipp, A. L. , … Sytsma, K. J. (2009). Origin, adaptive radiation and diversification of the Hawaiian lobeliads (Asterales: Campanulaceae). Proceedings of the Royal Society B‐Biological Sciences, 276, 407–416. 10.1098/rspb.2008.1204 PMC2664350 18854299

[jbi13525-bib-0029] Goodall‐Copestake, W. P. , Harris, D. J. , & Hollingsworth, P. M. (2009). The origin of a mega‐diverse genus: Dating *Begonia* (Begoniaceae) using alternative datasets, calibrations and relaxed clock methods. Botanical Journal of the Linnean Society, 159, 363–380. 10.1111/j.1095-8339.2009.00948.x

[jbi13525-bib-0030] Hassemer, G. , De Giovanni, R. , & Trevisan, R. (2016). The use of potential distribution models in the study of the distribution and conservation status of plants: The case of *Plantago* L. (Plantaginaceae) in Brazil. Journal of the Torrey Botanical Society, 143, 38–49. 10.3159/TORREY-D-14-00070

[jbi13525-bib-0031] Hassemer, G. , Moroni, P. , & O'Leary, N. (2018). A nomenclatural revision of *Littorella* (Plantaginaceae, Plantagineae). Taxon, 67, 1024–1028. 10.12705/675.14

[jbi13525-bib-0032] Heleno, R. , & Vargas, P. (2015). How do islands become green? Global Ecology and Biogeography, 24, 518–526. 10.1111/geb.12273

[jbi13525-bib-0033] Hennequin, S. , Kessler, M. , Lindsay, S. , & Schneider, H. (2014). Evolutionary patterns in the assembly of fern diversity on the oceanic Mascarene Islands. Journal of Biogeography, 41, 1651–1663. 10.1111/jbi.12339

[jbi13525-bib-0034] Henshaw, H. W. (1910). Migration of the Pacific Plover to and from the Hawaiian Islands. The Auk, 27, 245–262. 10.2307/4071308

[jbi13525-bib-0035] Hipsley, C. A. , & Müller, J. (2014). Beyond fossil calibrations: Realities of molecular clock practices in evolutionary biology. Frontiers in Genetics, 5, 706–11. 10.3389/fgene.2014.00138 PMC4033271 24904638

[jbi13525-bib-0036] Ho, S. Y. W. , Tong, K. J. , Foster, C. S. P. , Ritchie, A. M. , Lo, N. , & Crisp, M. D. (2015). Biogeographic calibrations for the molecular clock. Biology Letters, 11, 20150194 10.1098/rsbl.2015.0194 26333662 PMC4614420

[jbi13525-bib-0037] Hoggard, R. K. , Kores, P. J. , Molvray, M. , Hoggard, G. D. , & Broughton, D. A. (2003). Molecular systematics and biogeography of the amphibious genus *Littorella* (Plantaginaceae). American Journal of Botany, 90, 429–435. 10.3732/ajb.90.3.429 21659136

[jbi13525-bib-0038] Hultén, E. (1930). Flora of Kamtchatka and the adjacent islands (Vol. 4). Stockholm: Almqvist & Wiksell.

[jbi13525-bib-0039] Ishikawa, N. , Yokoyama, J. , Ikeda, H. , Takabe, E. , & Tsukaya, H. (2009). Evaluation of morphological and molecular variation in *Plantago asiatica* var. *densiuscula*, with special reference to the systematic treatment of *Plantago asiatica* var. *yakusimensis* . Journal of Plant Research, 119, 385–395. 10.1007/s10265-006-0286-y 16773281

[jbi13525-bib-0040] Jenni, L. , & Jenni‐Eiermann, S. (1998). Fuel supply and metabolic constraints in migrating birds. Journal of Avian Biology, 29, 521–528. 10.2307/3677171

[jbi13525-bib-0041] Johnson, M. A. , Clark, J. R. , Wagner, W. L. , & McDade, L. A. (2017). A molecular phylogeny of the Pacific Glade of *Cyrtandra* (Gesneriaceae) reveals a Fijian origin, recent diversification, and the importance of founder events. Molecular Phylogenetics and Evolution, 116, 30–48. 10.1016/j.ympev.2017.07.004 28705455

[jbi13525-bib-0042] Kainulainen, K. , Razafimandimbison, S. G. , Wikstrom, N. , & Bremer, B. (2017). Island hopping, long‐distance dispersal and species radiation in the Western Indian Ocean: Historical biogeography of the Coffeeae alliance (Rubiaceae). Journal of Biogeography, 44, 1966–1979. 10.1111/jbi.12981

[jbi13525-bib-0103] Katoh, K. , & Standley, D. M. (2013). MAFFT multiple sequence alignment software version 7: Improvements in performance and usability. Molecular Biology and Evolution, 30, 772–780. 23329690 10.1093/molbev/mst010 PMC3603318

[jbi13525-bib-0043] Kearse, M. , Moir, R. , Wilson, A. , Stones‐Havas, S. , Cheung, M. , Sturrock, S. , … Drummond, A. (2012). Geneious Basic: An integrated and extendable desktop software platform for the organization and analysis of sequence data. Bioinformatics, 28, 1647–1649. 10.1093/bioinformatics/bts199 22543367 PMC3371832

[jbi13525-bib-0044] Kistler, L. , Montenegro, A. , Smith, B. D. , Gifford, J. A. , Green, R. E. , Newsom, L. A. , & Shapiro, B. (2014). Transoceanic drift and the domestication of African bottle gourds in the Americas. Proceedings of the National Academy of Sciences of the United States of America, 111, 2937–2941. 10.1073/pnas.1318678111 24516122 PMC3939861

[jbi13525-bib-0045] Kolář, J. (2014). *Littorella uniflora* (L.) Ascherson: A review. Scientia Agriculturae Bohemica, 45, 147–154.

[jbi13525-bib-0046] Kreitschitz, A. , Kovalev, A. , & Gorb, S. N. (2016). “Sticky invasion”—The physical properties of *Plantago lanceolata* L. seed mucilage. Beilstein Journal of Nanotechnology, 7, 1918–1927. 10.3762/bjnano.7.183 28144540 PMC5238637

[jbi13525-bib-0047] Landis, M. J. , Matzke, N. J. , Moore, B. R. , & Huelsenbeck, J. P. (2013). Bayesian analysis of biogeography when the number of areas is large. Systematic Biology, 62, 789–804. 10.1093/sysbio/syt040 23736102 PMC4064008

[jbi13525-bib-0048] le Roux, J. J. , Strasberg, D. , Rouget, M. , Morden, C. W. , Koordom, M. , & Richardson, D. M. (2014). Relatedness defies biogeography: The tale of two island endemics (*Acacia heterophylla* and *A. koa*). New Phytologist, 204, 230–242. 10.1111/nph.12900 24942529

[jbi13525-bib-0049] Losos, J. B. , & Ricklefs, R. E. (2009). Adaptation and diversification on islands. Nature, 457, 830–836. 10.1038/nature07893 19212401

[jbi13525-bib-0051] Matzke, N.J. (2013). BioGeoBEARS: BioGeography with Bayesian (and likelihood) evolutionary analysis in R Scripts, CRAN: The Comprehensive R Archive Network, Vienna, Austria. http://cran.r-project.org/package=BioGeoBEARS.

[jbi13525-bib-0052] Matzke, N. J. (2014). Model selection in historical biogeography reveals that founder‐event speciation is a crucial process in island clades. Systematic Biology, 63, 951–970. 10.1093/sysbio/syu056 25123369

[jbi13525-bib-0053] McCormick, M. K. , & Jacquemyn, H. (2014). What constrains the distribution of orchid populations? New Phytologist, 202, 392–400. 10.1111/nph.12639

[jbi13525-bib-0054] McDougall, I. , Embleton, B. J. J. , & Stone, D. B. (1981). Origin and evolution of Lord‐Howe Island, southwest Pacific Ocean. Journal of the Geological Society of Australia, 28, 155–176. 10.1080/00167618108729154

[jbi13525-bib-0055] Meudt, H. M. (2011). Amplified fragment length polymorphism data reveal a history of auto and allopolyploidy in New Zealand endemic species of *Plantago* (Plantaginaceae): New perspectives on a taxonomically challenging group. International Journal of Plant Sciences, 172(220), 237.

[jbi13525-bib-0056] Meudt, H. M. (2012). A taxonomic revision of native New Zealand *Plantago* (Plantaginaceae). New Zealand Journal of Botany, 50, 101–178. 10.1080/0028825X.2012.671179

[jbi13525-bib-0057] Missouri Botanical Garden and Harvard University Herbaria (2017). eFloras. Retrieved from http://www.efloras.org (Accessed: 10th February 2017)

[jbi13525-bib-0058] Mitchell, T. C. , Williams, B. R. M. , Wood, J. R. I. , Harris, D. J. , Scotland, R. W. , & Carine, M. A. (2016). How the temperate world was colonised by bindweeds: Biogeography of the Convolvuleae (Convolvulaceae). BMC Evolutionary Biology, 16, 16 10.1186/s12862-016-0591-6 26787507 PMC4719731

[jbi13525-bib-0102] Mueller, J. (1981). Fossil pollen records of extant angiosperms. Botanical Review, 47, 706–142.

[jbi13525-bib-0059] Nathan, R. (2006). Long‐distance dispersal of plants. Science, 313, 786–788. 10.1126/science.1124975 16902126

[jbi13525-bib-0061] Nathan, R. , Schurr, F. M. , Spiegel, O. , Steinitz, O. , Trakhtenbrot, A. , & Tsoar, A. (2008). Mechanisms of long‐distance seed dispersal. Trends in Ecology & Evolution, 23, 638–647. 10.1016/j.tree.2008.08.003 18823680

[jbi13525-bib-0062] Nogales, M. , Heleno, R. , Traveset, A. , & Vargas, P. (2012). Evidence for overlooked mechanisms of long‐distance seed dispersal to and between oceanic islands. New Phytologist, 194, 313–317. 10.1111/j.1469-8137.2011.04051.x 22250806

[jbi13525-bib-0063] Oxelman, B. , Lidén, M. , & Berglund, D. (1997). Chloroplast *rps16* intron phylogeny of tribe *Sileneae* (Caryophyllaceae). Plant Systematics and Evolution, 206, 393–410. 10.1007/BF00987959

[jbi13525-bib-0064] Panter, C. J. , & Dolman, P. M. (2012). Mammalian herbivores as potential seed dispersal vectors in ancient woodland fragments. Wildlife Biology, 18, 292–303. 10.2981/11-112

[jbi13525-bib-0065] Patzak, A. , & Rechinger, K. H. (1965). Plantaginaceae In Rechinger K. H. (Ed.), Flora Iranica (23 pp). Vienna, Austria: Naturhistorisches Museum.

[jbi13525-bib-0066] Pilger, R. (1937). Plantaginaceae In Engler A. (Ed.), Das Pflanzenreich IV 269 (102, Heft). Leipzig: Wilhelm Engelmann.

[jbi13525-bib-0067] Pole, M. (1994). The New‐Zealand Flora—Entirely long‐distance dispersal. Journal of Biogeography, 21, 625–635. 10.2307/2846036

[jbi13525-bib-0068] Rahn, K. (1996). A phylogenetic study of the Plantaginaceae. Botanical Journal of the Linnean Society, 120, 145–198.

[jbi13525-bib-0069] Rambaut, A. (2012). FigTree v1.4. Retrieved from http://tree.bio.ed.ac.uk/software/figtree.

[jbi13525-bib-0070] Rambaut, A. , Suchard, M.A. , Xie, D. , & Drummond, A. (2014). Tracer v1.6. Retrieved from http://beast.bio.ed.ac.uk/Tracer.

[jbi13525-bib-0071] Raxworthy, C. J. , Forstner, M. R. J. , & Nussbaum, R. A. (2002). Chameleon radiation by oceanic dispersal. Nature, 415, 784–787. 10.1038/415784a 11845207

[jbi13525-bib-0072] Ree, R. H. , & Smith, S. A. (2008). Maximum Likelihood inference of geographic range evolution by dispersal, local extinction, and cladogenesis. Systematic Biology, 57, 4–14. 10.1080/10635150701883881 18253896

[jbi13525-bib-0073] Richardson, J. E. , Weitz, F. M. , Fay, M. F. , Cronk, Q. C. B. , Linder, H. P. , Reeves, G. , & Chase, M. W. (2001). Phylogenetic analysis of *Phylica* L. with an emphasis on island species: Evidence from plastid *trnL‐F* DNA and nuclear internal transcribed spacer (ribosomal DNA) sequences. Taxon, 50, 405–427. 10.2307/1223889

[jbi13525-bib-0074] Ronquist, F. (1997). Dispersal‐Vicariance Analysis: A new approach to the quantification of historical biogeography. Systematic Biology, 46, 195–203. 10.1093/sysbio/46.1.195

[jbi13525-bib-0075] Ronquist, F. , & Huelsenbeck, J. P. (2003). mrbayes 3: Bayesian phylogenetic inference under mixed models. Bioinformatics, 19, 1572–1574. 10.1093/bioinformatics/btg180 12912839

[jbi13525-bib-0076] Rønsted, N. , Chase, M. W. , Albach, D. C. , & Bello, M. A. (2002). Phylogenetic relationships within *Plantago* (Plantaginaceae): Evidence from nuclear ribosomal ITS and plastid *trnL‐F* sequence data. Botanical Journal of the Linnean Society, 139, 323–338. 10.1046/j.1095-8339.2002.00070.x

[jbi13525-bib-0077] Sanmartin, I. , & Ronquist, F. (2004). Southern hemisphere biogeography inferred by event‐based models: Plant versus animal patters. Systematic Biology, 53, 216–243. 10.1080/10635150490423430 15205050

[jbi13525-bib-0078] Shaw, J. , Lickey, E. B. , Schilling, E. E. , & Small, R. L. (2007). Comparison of whole chloroplast genome sequences to choose noncoding regions for phylogenetic studies in angiosperms: The tortoise and the hare III. American Journal of Botany, 94, 275–288. 10.3732/ajb.94.3.275 21636401

[jbi13525-bib-0079] Simmons, M. P. , & Ochoterena, H. (2000). Gaps as characters in sequence‐based phylogenetic analyses. Systematic Biology, 49, 369–381. 10.1093/sysbio/49.2.369 12118412

[jbi13525-bib-0080] Stamatakis, A. (2014). RAxML version 8: A tool for phylogenetic analysis and post‐analysis of large phylogenies. Bioinformatics, 30, 1312–1313. 10.1093/bioinformatics/btu033 24451623 PMC3998144

[jbi13525-bib-0081] Stuessy, T. F. , Crawford, D. J. , & Ruiz, E. A. (2018). Patterns of phylogeny In Stuessy T. F., Crawford D. J., Loppez‐Sepulveda P., Baeza C. M., & Ruiz E. A. (Eds.), Plants of Oceanic Islands: Evolution, biogeography, and conservation of the flora or the Juan Fernández (Robinson Crusoe) Archipelago. Cambridge, United Kingdom: Cambridge University Press.

[jbi13525-bib-0082] Stuessy, T. F. , Foland, K. A. , Sutter, J. F. , & Silva, M. (1984). Botanical and geological significance of potassium‐argon dates from the Juan Fernández islands. Science, 225, 49–51. 10.1126/science.225.4657.49 17775659

[jbi13525-bib-0083] Sun, Y. , Skinner, D. Z. , Liang, G. H. , & Hulbert, S. H. (1994). Phylogenetic analysis of *Sorghum* and related taxa using internal transcribed spacers of nuclear ribosomal DNA. Theoretical and Applied Genetics, 89, 26–32. 10.1007/BF00226978 24177765

[jbi13525-bib-0084] Taberlet, P. , Gielly, L. , Pautou, G. , & Bouvet, J. (1991). Universal primers for amplification of three non‐coding regions of chloroplast DNA. Plant Molecular Biology, 17, 1105–1109. 10.1007/BF00037152 1932684

[jbi13525-bib-0085] Tay, M. L. , Meudt, H. M. , Garnock‐Jones, P. J. , & Ritchie, P. A. (2010a). DNA sequences from three genomes reveal multiple long‐distance dispersals and non‐monophyly of sections in Australasian *Plantago* (Plantaginaceae). Australian Systematic Botany, 23, 47–68. 10.1071/SB09040

[jbi13525-bib-0086] Tay, M. L. , Meudt, H. M. , Garnock‐Jones, P. J. , & Ritchie, P. A. (2010b). Testing species limits of New Zealand *Plantago* (Plantaginaceae) using internal transcribed spacer (ITS) DNA sequences. New Zealand Journal of Botany, 48(205), 224.

[jbi13525-bib-0087] van der Aart, P. J. M. , & Vulto, J. C. (1992). General biology of *Plantago*. Biogeography and human effects In Kuiper P. J. C., & Bos M. (Eds.), Plantago: A multidisciplinary study (pp. 5–6)., *Ecological Studies*,** 89** Paris: Springer‐Verlag.

[jbi13525-bib-0088] Vences, M. , Vieites, D. R. , Glaw, F. , Brinkmann, H. , Kosuch, J. , Veith, M. , & Meyer, A. (2003). Multiple overseas dispersal in amphibians. Proceedings of the Royal Society B‐Biological Sciences, 270, 2435–2442. 10.1098/rspb.2003.2516 PMC1691525 14667332

[jbi13525-bib-0089] Viana, D. S. , Gangoso, L. , Bouten, W. , & Figuerola, J. (2016). Overseas seed dispersal by migratory birds. Proceedings of the Royal Society B‐Biological Sciences, 283. 10.1098/rspb.2015.2406 PMC4721096 26740610

[jbi13525-bib-0090] Wagner, W. L. , Herbst, D. R. , & Sohmer, S. H. (1990). Manual of flowering plants of Hawaii. Honolulu, Hawaii, USA: University of Hawaii Press and Bishop Museum Press.

[jbi13525-bib-0091] Western, T. L. (2012). The sticky tale of seat coat mucilages: Production, genetics, and role in seed germination and dispersal. Seed Science Research, 22, 706–25. 10.1017/S0960258511000249

[jbi13525-bib-0092] Winkworth, R. C. , Wagstaff, S. J. , Glenny, D. , & Lockhart, P. J. (2002). Plant dispersal N.E.W.S from New Zealand. Trends in Ecology and Evolution, 17, 514–520. 10.1016/S0169-5347(02)02590-9

[jbi13525-bib-0094] Zielske, S. , Ponder, W. F. , & Haase, M. (2017). The enigmatic pattern of long‐distance dispersal of minute freshwater gastropods (Caenogastropoda, Truncatelloidea, Tateidae) across the South Pacific. Journal of Biogeography, 44, 195–206. 10.1111/jbi.12800

