# Supplementary material for: Ancestral range reconstruction of remote oceanic island species of Plantago (Plantaginaceae) reveals differing scales and modes of dispersal
Source: J Biogeogr. 2019 Mar 15;46(4):706–22. doi: 10.1111/jbi.13525 (PMC6559316; doi:10.1111/jbi.13525)
Supplement: Supplementary file 1 [file JBI-46-706-s001.docx]

***Journal of Biogeography***

**SUPPORTING INFORMATION**

**Ancestral range reconstruction of remote oceanic island species of *Plantago*** **(Plantaginaceae)** **reveals differing scales and modes of dispersal.**

N. Iwanycki Ahlstrand, B. Verstraete, G. Hassemer, S. Dunbar-Co, R. Hoggard, H.M. Meudt, N. Rønsted.

**Appendix I**. List of oceanic island taxa known from subgenus *Plantago* L. The fourteen species represented in the current study are listed in bold, and an asterisk (*) follows the species name. Taxa from all islands and island systems regardless of their geological histories are included, except for taxa from mainland Australia.

| Species | Native distribution | Sect. in Rahn (1996) | Included in past phylogenetic studies |
| --- | --- | --- | --- |
| *P. alata* Nakai | Jeju | *Plantago* |  |
| *P. aucklandica* Hook.f. * | Auckland Islands | *Plantago* | Tay et al. (2010a); Tay et al. (2010b); Meudt (2011); Meudt (2012) |
| *P. aundensis* P.Royen | New Guinea | *Oliganthos* |  |
| *P. bellidioides* Decne. | Tasmania | *Mesembrynia* |  |
| *P. coreana* H.Lév. | Jeju | *Plantago* |  |
| *P. daltonii* Decne. | Tasmania | *Mesembrynia* | Hoggard et al. (2003); Tay et al. (2010a) |
| *P. depauperata* Merr. & L.M.Perry | New Guinea | *Oliganthos* |  |
| *P. euana* Hurlim. | Tonga Islands | *Mesembrynia* |  |
| *P. fernandezia* Bertero ex Barnéoud * | Juan Fernández Islands | *Plantago* |  |
| *P. galapagensis* Rahn | Galápagos Islands | *Virginica* |  |
| *P. glabrata* Hook.f. | Tasmania | *Mesembrynia* |  |
| *P. gunnii* Hook.f. | Tasmania | *Oliganthos* |  |
| *P. hakusanensis* Koidz. | Japan | *Plantago* |  |
| *P. hasskarlii* Decne. | Java | *Plantago* |  |
| *P. hawaiensis* (A.Gray) Pilg. * | Hawaii Islands | *Plantago* | Dunbar-Co et al. (2008) |
| *P. hedleyi* Maiden * | Lord Howe Island | *Plantago* |  |
| *P. incisa* Hassk. * | Java | *Plantago* |  |
| *P. lanigera* Hook.f. | New Zealand | *Oliganthos* | Tay et al. (2010a); Tay et al. (2010b); Meudt (2011); Meudt (2012) |
| *P. montisdicksonii* P.Royen | New Guinea | *Mesembrynia* |  |
| *P. moorei* Rahn * | West Falkland | *Oliganthos* |  |
| *P. novae-zelandiae* L.B.Moore | New Zealand | *Oliganthos* | Tay et al. (2010b); Meudt (2011); Meudt (2012) |
| *P. obconica* Sykes | New Zealand | *Oliganthos* | Tay et al. (2010a); Tay et al. (2010b); Meudt (2011); Meudt (2012 |
| *P. pachyphylla* A.Gray * | Hawaii Islands | *Plantago* | Dunbar-Co et al. (2008) |
| *P. papuana* P.Royen | New Guinea | *Mesembrynia* |  |
| *P. paradoxa* Hook.f. * | Tasmania | *Oliganthos* | Hoggard et al. (2003); Tay et al. (2010a) |
| *P. pentasperma* Hemsl. | St. Paul and New Amsterdam Islands | *Mesembrynia* |  |
| *P. picta* Colenso | New Zealand | *Mesembrynia* | Tay et al. (2010b); Meudt (2011); Meudt (2012) |
| *P. polita* Craven | New Guinea | *Oliganthos* |  |
| *P. princeps* Cham. & Schltdl. * | Hawaii Islands | *Plantago* | Dunbar-Co et al. (2008) |
| *P. raoulii* Decne. * | New Zealand | *Mesembrynia* | Rønsted et al. (2002); Ishikawa et al. (2009); Tay et al. (2010a); Tay et al. (2010b); Meudt (2011); Meudt (2012) |
| *P. rapensis* Pilg. * | Rapa Iti Island | *Plantago* | Dunbar-Co et al. (2008) |
| *P. robusta* Roxb. | Saint Helena Island | *Plantago* |  |
| *P. rupicola* Pilg. * | Rapa Iti Island | *Plantago* | Dunbar-Co et al. (2008) |
| *P. spathulata* Hook.f. | New Zealand | *Mesembrynia* | Rønsted et al. (2002); Ishikawa et al. (2009); Tay et al. (2010a); Tay et al. (2010b); Meudt (2011); Meudt (2012) |
| *P. stauntonii* Reichardt * | St. Paul and New Amsterdam Islands | *Mesembrynia* | Rønsted et al. (2002); Ishikawa et al. (2009) |
| *P. stenophylla* Merr. & L.M.Perry | New Guinea | *Oliganthos* |  |
| *P. tanalensis* Baker | Madagascar | *Plantago* |  |
| *P. taquetii* H.Lév. * | Jeju | *Plantago* |  |
| *P. tasmanica* Hook.f. | Tasmania | *Mesembrynia* | Hoggard et al. (2003); Tay et al. (2010a) |
| *P. triandra* Berggr. | New Zealand | *Oliganthos* | Hoggard et al. (2003); Tay et al. (2010a); Tay et al. (2010b); Meudt (2011); Meudt (2012) |
| *P. triantha* Spreng. | Auckland Islands and Tasmania | *Oliganthos* | Tay et al. (2010a); Tay et al. (2010b); Meudt (2011); Meudt (2012) |
| *P. trichophora* Merr. & L.M.Perry | New Guinea | *Mesembrynia* |  |
| *P. trinitatis* Rahn | Trindade Island | *Virginica* | Rønsted et al. (2002); Ishikawa et al. (2009) |
| *P. udicola* Meudt & Garn.-Jones | New Zealand | none (new species) |  |
| *P. unibracteata* Rahn | New Zealand | *Oliganthos* |  |

**References**

Dunbar-Co, S., Wieczorek, A.M., & Morden, C.W. (2008). Molecular phylogeny and adaptive radiation of the endemic Hawaiian *Plantago* species (Plantaginaceae). *American Journal of Botany*, **95**, 1177-1188.

Hoggard, R.K., Kores, P.J., Molvray, M., Hoggard, G.D., & Broughton, D.A. (2003). Molecular systematics and biogeography of the amphibious genus *Littorella* (Plantaginaceae). *American Journal of Botany*, **90**, 429-435.

Ishikawa, N., Yokoyama, J., Ikeda, H., Takabe, E., & Tsukaya, H. (2006). Evaluation of morphological and molecular variation in *Plantago asiatica* var. *densiuscula*, with special reference to the systematic treatment of *Plantago asiatica* var. *yakusimensis*. *Journal of Plant Research*, **119**, 385-395.

Meudt, H.M. (2011). Amplified fragment length polymorphism data reveal a history of auto and allopolyploidy in New Zealand endemic species of *Plantago* (Plantaginaceae): New perspectives on a taxonomically challenging group. *International Journal of Plant Sciences*, **172**, 220 237.

Meudt, H.M. (2012). A taxonomic revision of native New Zealand *Plantago* (Plantaginaceae). *New Zealand Journal of Botany*, **50**, 101-178.

Rønsted, N., Chase, M.W., Albach, D.C., & Bello, M.A. (2002). Phylogenetic relationships within *Plantago* (Plantaginaceae): evidence from nuclear ribosomal ITS and plastid *trnL-F* sequence data. *Botanical Journal of the Linnean Society*, **139**, 323-338.

Tay, M.L., Meudt, H.M., Garnock-Jones, P.J., & Ritchie, P.A. (2010a). DNA sequences from three genomes reveal multiple long-distance dispersals and non-monophyly of sections in Australasian *Plantago* (Plantaginaceae). *Australian Systematic Botany*, **23**, 47-68.

Tay M.L., Meudt H.M., Garnock-Jones P.J, Ritchie P.A (2010b). Testing species limits of New Zealand *Plantago* (Plantaginaceae) using internal transcribed spacer (ITS) DNA sequences. *New Zealand Journal of Botany* **48**, 205 224.

**Appendix II.** List of DNA regions and primer details used in the study of *Plantago* section *Plantago*. Model results from jModeltest2 are included.

| Marker | Primer name and sequence | Model | Reference |
| --- | --- | --- | --- |
| ITS | 17SE: ACG AAT TCA TGG TCC GGT GAA GTG TTC G  26SE: TAG AAT TCC CCG GTT CGC TCG CCG TTA C | SYM+G | Rønsted et al., 2002; Sun et al., 1994. |
| *rps16* | 1F: GTG GTA GAA AGC AAC GTG CGA CTT  2R: TCG GGA TCG AAC ATC AAT TGC AAC | GTR+G | Oxelman et al., 1997. |
| *trnLF* | c: CGA AAT CGG TAG ACG CTA CG  f: ATT TGA ACT GGT GAC ACG AG | TVM+G | Rønsted et al., 2002; Taberlet et al., 1991. |
| *ndhF-rpl32* | rpL32-R: CCA ATA TCC CTT YYT TTT CCA A  ndhF: GAA AGG TAT KAT CCA YGM ATA TT | TVM+G | Dunbar-Co et al., 2008; Shaw et al., 2007. |
| *rpl32-trnL* | trnL^(UAG)^: CTG CTT CCT AAG AGC AGC GT  rpL32-F: CAG TTC CAA AAA AAC GTA CTT C | TVM+G | Dunbar-Co et al., 2008; Shaw et al., 2007. |

**References**

Dunbar-Co, S., Wieczorek, A.M., & Morden, C.W. (2008). Molecular phylogeny and adaptive radiation of the endemic Hawaiian *Plantago* species (Plantaginaceae). *American Journal of Botany*, **95**, 1177-1188.

Oxelman, B., Lidén, M., & Berglund, D. (1997). Chloroplast *rps16* intron phylogeny of tribe *Sileneae* (Caryophyllaceae). *Plant Systematics and Evolution,* **206,** 393–410.

Rønsted, N., Chase, M.W., Albach, D.C., & Bello, M.A. (2002). Phylogenetic relationships within *Plantago* (Plantaginaceae): evidence from nuclear ribosomal ITS and plastid *trnL-F* sequence data. *Botanical Journal of the Linnean Society*, **139**, 323-338.

Shaw, J, Lickey, EB, Schilling, EE, & Small, RL. (2007). Comparison of whole chloroplast genome sequences to choose noncoding regions for phylogenetic studies in angiosperms: The tortoise and the hare III. *American Journal of Botany,* **94,** 275–288.

Sun, Y., Skinner, D.Z., Liang, G.H., & Hulbert, S.H. (1994). Phylogenetic analysis of *Sorghum* and related taxa using internal transcribed spacers of nuclear ribosomal DNA. *Theoretical and Applied Genetics,* **89,** 26–32.

Taberlet, P., Gielly, L., Pautou, G., & Bouvet, J. (1991). Universal primers for amplification of three non-coding regions of chloroplast DNA. *Plant Molecular Biology,* **17,** 1105–1109.

**Figure S1**. The 50% majority rule consensus cladogram from the MrBayes analysis for ITS sequences for members from subgenus *Plantago* L. The posterior probabilities are listed at the nodes.

**Figure S2**. The 50% majority rule consensus cladogram from the MrBayes analysis for plastid sequences (*trnlF*, *rps16, ndhF*–*rpl32* and *rpl32*–*trnL*) for members from subgenus *Plantago* L. The posterior probabilities are listed at the nodes.

**
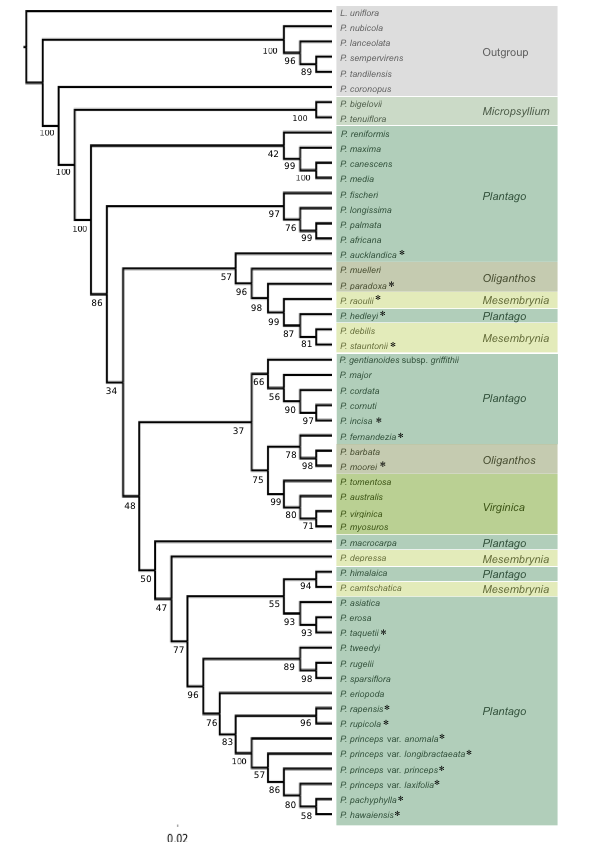
**

**Figure S3**. Maximum likelihood cladogram from the RAxML analysis for nuclear (ITS) and plastid sequences (*trnlF*, *rps16, ndhF*–*rpl32* and *rpl32*–*trnL*) for members from subgenus *Plantago* L. Bootstrap support values are listed at the nodes.
